# Inhibition of tenascin C rescues abnormally reduced Na currents in dystrophin-deficient ventricular cardiomyocytes

**DOI:** 10.1152/ajpheart.00307.2025

**Published:** 2025-08-11

**Authors:** Jessica Marksteiner, Jakob Sauer, Martin Hohenegger, Faith-Oluwakemi David, Natalie Schindler, Petra Lujza Szabó, Attila Kiss, Christopher Dostal, Bruno K. Podesser, Elena Lilliu, Benjamin Hackl, Hannes Todt, Xaver Koenig, Karlheinz Hilber, Klaus Schicker

**Affiliations:** 1 Department of Neurophysiology and Neuropharmacology, Center for Physiology and Pharmacology, Medical University of Vienna, Vienna, Austria; 2 Institute of Pharmacology, Center for Physiology and Pharmacology, Medical University of Vienna, Vienna, Austria; 3 Ludwig Boltzmann Institute for Cardiovascular Research at the Center for Biomedical Research and Translational Surgery, Medical University of Vienna, Vienna, Austria

**Keywords:** arrhythmias, cardiomyopathy, Duchenne muscular dystrophy, Na current, tenascin C

## Abstract

Cardiac arrhythmias significantly contribute to mortality in Duchenne muscular dystrophy (DMD), a severe muscle disease caused by dystrophin deficiency. Using the *mdx* mouse model for human DMD, we previously showed that the lack of dystrophin induces a significant loss of peak sodium current (*I*_Na_) in ventricular cardiomyocytes. This provided a mechanistic explanation for ventricular conduction defects and concomitant arrhythmias in the dystrophic heart. The extracellular matrix protein tenascin C (TN-C), a major remodeling factor in the diseased heart, is strongly upregulated in DMD. The consequences of TN-C upregulation in the dystrophic heart, however, are unknown. Here, we tested if TN-C induces electrical remodeling in the dystrophic heart, and if inhibition of TN-C rescues peak I_Na_ loss in dystrophin-deficient ventricular cardiomyocytes. We found that cardiomyocytes from TN-C knockout (KO) mice had increased peak *I*_Na_. The abnormally reduced peak *I*_Na_ in *mdx* myocytes was rescued to wild-type levels by additional TN-C KO, which was accompanied by enhanced Na_v_1.5 channel expression. Further, peak *I*_Na_ in *mdx* myocytes was increased by treatment of *mdx* mice with TN-C siRNA. Twenty-four-hour incubation of wild-type myocytes with human recombinant TN-C reduced their peak *I*_Na_, an effect which could be abolished by blocking antibodies specific for the α-7 integrin subunit. Our findings suggest that TN-C induces peak *I*_Na_ loss in the dystrophic heart, and that inhibition of TN-C expression rescues abnormally reduced peak *I*_Na_ in dystrophin-deficient ventricular cardiomyocytes. TN-C inhibition emerges as a strategy to counteract ventricular conduction impairments and arrhythmias in patients with DMD.

## Introduction

Dilated cardiomyopathy development and cardiac arrhythmias significantly contribute to mortality in Duchenne muscular dystrophy (DMD), a fatal disease caused by dystrophin deficiency (1, 2). A significant source of arrhythmia vulnerability in patients with DMD is slowed ventricular impulse conduction (3, 4), which predisposes to ventricular asynchrony and the development of reentrant circuits. Using the dystrophin-deficient *mdx* mouse model for human DMD (5), we (6–8) and others (9–12) previously reported that the lack of dystrophin causes a significant peak sodium current (*I*_Na_) loss in ventricular cardiomyocytes. Peak *I*_Na_ was also decreased in induced pluripotent stem cell-derived cardiomyocytes from patients with DMD (13). These findings provided a mechanistic explanation for ventricular conduction delay and concomitant arrhythmias in the dystrophic heart.

Tenascin C (TN-C) is an extracellular matrix (ECM) protein involved in important biological signaling pathways in the course of physiological and pathological tissue remodeling. In the heart, TN-C appears in a temporally restricted manner during development, but is hardly detectable in adult cardiac tissue. Under pathological conditions, in the injured and diseased heart, however, TN-C is re-expressed and considered a significant remodeling factor causing mostly detrimental effects (14–20). TN-C, mainly produced by cardiac fibroblasts, is also released into the bloodstream in the course of a wide range of cardiac disease conditions in humans, and used as a biomarker predicting the degree of cardiac remodeling and subsequent mortality in patients (14–17, 21).

Very little is known about the role of TN-C in the dystrophic heart. In patients with DMD (22) and *mdx* mice (23), serum TN-C levels are increased. In addition, in *mdx* mice, plasma levels of TN-C are positively correlated with left ventricular dilation (23). Enhanced TN-C protein levels have also been detected in ventricular tissue from dystrophic mice and rats (24, 25), but the protein’s effects in the dystrophic heart are unknown. Possible effects of TN-C on cardiac electrophysiological properties in the diseased heart in general (i.e., the protein’s potential to trigger “electrical remodeling”), and in dystrophic cardiomyopathy in particular, have not been investigated so far.

In the present study, we explored the hypotheses that TN-C contributes to electrical remodeling in the dystrophic heart and that inhibition of TN-C rescues peak *I*_Na_ loss in dystrophin-deficient ventricular cardiomyocytes.

## Methods

### Ethical Approval

The investigation coincided with the rules of the Animal Welfare Committee of the Medical University of Vienna and conformed to the guiding principles of the Declaration of Helsinki. The experimental protocols were approved by the Austrian Science Ministry. The respective ethics vote has the following number: BMWFW-66.009/0175-WF/V/3b/2015.

### Animal Models

Mice in an age range between 12 and 20 wk were used in this study. Only male mice were used because of the X-linked inheritance of DMD and potential translational relevance to human patients, as in Haffner et al. (26). Dystrophin-deficient *mdx* mice on the BL10 background (C57BL/10ScSn-Dmdmdx/J) and wild-type (wt) control mice (C57BL/10ScSnJ) originated from Charles River Laboratories. Tenascin-C (TN-C) knockout (KO) mice originated from KO, RBRC00007 A, Experimental Animal Division, Tsukuba, Japan (27, 28). This strain was crossed with *mdx* mice to obtain *mdx*-TN-C double KO mice. Nine-month-old male dystrophin-deficient *DMD*^mdx^ Sprague–Dawley rats (29) originated from INSERM-CRTI UMR 1064 (Nantes). Age- and sex-matched control wt Sprague–Dawley rats originated from Janvier Laboratories. Genotyping of mice and rats was performed using standard PCR assays.

### siRNA Treatment Procedure In Vivo

Tenascin-C (TNC) gene expression was silenced in *mdx* mice using small interfering RNA (siRNA). The transfection protocol was initiated at 6 wk of age and continued for 9 wk, with intraperitoneal administration of the siRNA complex or vehicle (PBS) twice weekly. The complexation procedure followed the methodology described by Fu et al. (30). Briefly, 100 μg of TNC-specific siRNA [Tenascin-C siRNA (h); sc-43186, Santa Cruz Biotechnology, Inc.] was complexed with in vivo-jetPEI transfection reagent (Polyplus-transfection, Inc.) according to the manufacturer’s protocol. Supplemental Fig. S1 proves the effectiveness of the used siRNA to reduce TN-C levels.

### Isolation of Ventricular Cardiomyocytes and TN-C Incubation Experiments

Mice were anesthetized using isoflurane (2%, inhalation) and euthanized by cervical dislocation. Ventricular cardiomyocytes were then isolated using a Langendorff setup (Hugo Sachs Elektronik, March, Germany) according to the isolation procedure described in detail in our previous study (6). In brief, the hearts were rapidly removed, and a cannula was inserted into the aorta for retrograde perfusion with Ca-free solution containing 0.17 mg/mL Liberase TH (Roche) at 37°C for 18 min. Thereafter, both right and left ventricles were cut into pieces and incubated on a shaker at 37°C. The Ca^2+^ concentration was then increased to 150 μM over 30 min in four steps. Subsequently, pieces of digested tissue were triturated to liberate ventricular cardiomyocytes. After a centrifugation step, the myocytes were resuspended in minimum essential medium (MEM)-α (Sigma), containing ITS media supplement (Sigma) diluted (1:100), 2 mM L-glutamine, 100 U/mL penicillin, 0.1 mg/mL streptomycin, and 17 μM blebbistatin (Sigma). Finally, myocytes were plated on Matrigel (Becton Dickinson)-coated culture dishes.

For TN-C incubation experiments, acutely isolated ventricular cardiomyocytes were incubated with human recombinant TN-C (hTN-C, R&D Systems, 3358-TC-050, dissolved in ddH_2_O) for 24 h in culture medium at 37°C. Two different concentrations of TN-C were used: 30 ng/ML representing physiological serum levels (22, 31), and a significantly higher concentration of 1 μg/mL. The latter concentration was used because myocytes in the vicinity of TN-C-generating cardiac fibroblasts may be exposed to high local TN-C concentrations in a failing heart. In a separate series of experiments, integrin α-7 subunit-blocking antibodies (Abcam, ab195959, 1:500) were applied during 24 h TN-C incubation of cardiomyocytes, similar as in Ref. 32. Furthermore, integrin β-1 subunit-blocking antibodies (Cell Signaling, Cat. No. 3471, 1:500) and integrin α-5 sub-unit-blocking antibodies (Cell Signaling, Cat. No. 98204, 1:500) were also used.

### Sodium Current Recordings

The whole cell patch clamp method was used to record *I*_Na_ from ventricular cardiomyocytes up to 6 h after preparation (except for 24-h TN-C incubation experiments). All experiments were performed at room temperature (22 ± 1.5°C) by using an Axopatch 200B patch clamp amplifier (Axon Instruments, Union City, CA). Pipettes were formed from aluminosilicate glass (A120-77-10; Science Products, Hofheim, Germany) with a P-97 horizontal puller (Sutter Instruments, Novato, CA). They had resistances between 0.9 and 1.6 MΩ when filled with pipette solution. Data acquisition was carried out with pClamp 10 software (Axon Instruments) through a 16-bit A-D/D-A interface (Digidata1440; Axon Instruments). Data were low-pass filtered with 10 kHz (−3 dB) and digitized at 35 kHz. Data were analyzed with Clampfit 10.7 (Axon Instruments) and GraphPad Prism 8 (San Diego, CA) software. Current-voltage (*I*-*V*) relationships were fit with the function: *I = G*_max_·(*V* − *V*_rev_)/{ 1 + exp[(*V*_50_ − *V*)/*K*]}, where *I* is the current, *G*_max_ is the maximum conductance, *V* is the membrane potential, *V*_rev_ is the reversal potential, *V*_50_ is the voltage at which half-maximum activation occurred, and *K* is the slope factor. Membrane voltage was corrected for liquid junction potentials. For current density-voltage relationships, the current amplitudes at various potentials were measured and then divided by the membrane capacitance to yield current densities. The channels were activated by depolarizing voltage steps from a holding potential of −117 mV. The kinetics of fast inactivation were assessed by measuring the decay half-time, representing the time period between the current peak and the time point at which the current had decayed to 50%. Steady-state fast inactivation data were fit with the Boltzmann function: *I*/*I*_max_ 1/(1 + exp[(*V − V*_50_)/*K*)], where *I*/*I*_max_ is the normalized current, *V* is the membrane potential, *V*_50_ is the voltage at which half-maximum inactivation occurred, and *K* is the slope factor. Recordings from single ventricular cardiomyocytes were made in a bath solution that consisted of (in mM) 5 NaCl, 135 *N*-methyl-D-glucamine, 2.5 KCl, 1 CaCl_2_, 1 MgCl_2_, and 10 HEPES (pH 7.4), adjusted with HCl. The bath solution additionally contained 17 μM blebbistatin. The pipette solution contained (in mM) 5 NaCl, 110 CsF, 10 EGTA, and 10 HEPES (pH 7.3), adjusted with CsOH. A DAD-8-VC superfusion system (ALA Scientific Instruments, Westbury, NY) was used for continuous superfusion of actually recorded cells and allowed for rapid extracellular solution changes.

### RT-qPCR

The heart of the euthanized mouse was first perfused with 5 mL of ice-cold PBS while still in situ. Once excised, the heart was placed in a 6 cm dish containing ice-cold PBS, where the atria were removed, and the left ventricle was isolated. The left ventricle was then bisected, with one portion allocated for RT-qPCR and the other for Western blot analysis. For rat RT-qPCR analysis, the entire heart was used. The use of whole hearts for rat qPCR, but left ventricular tissue for mouse qPCR and Western blotting, may have impaired interspecies data comparability (also see Study Limitations). mRNA was extracted using the RNeasy mini kit (Qiagen) following the manufacturer’s instructions. The tissue was first lysed in buffer supplemented with β-mercaptoethanol, then homogenized using zirconia beads in a bead homogenizer. The concentration of the isolated mRNA was measured using a NanoDrop (Thermo Scientific), and cDNA synthesis was performed using the iScript reverse transcription supermix (BioRad). Real-time quantitative PCR was then conducted using the SsoAdvanced Universal SYBR Green Supermix (BioRad). Sample quantification was carried out on the StepOnePlus system (Applied Biosystems), and relative gene expression was determined using the 2^−ΔΔCt^ method. The following primers were used:

Tenascin-C mouse: FW 5′-GTTTGGAGACCGCCAGAGAAG-AA-3′, REV 5′-0TGTCCCCATATCTGCCCATCA-3′;

Tenascin-C rat: FW 5′-GGAGGTGACCATGCTGAGAT-3′, REV 5′-CTGTAAGTCCCTGGGTGCAT-3′;

*Scn5a* mouse: FW 5′-ATGGCAAACTTCCTGTTACCTC-3′, REV 5′-CCACGGGCTTGTTTTTCAGC-3′;

HPRT1 (housekeeper): Mouse FW 5′-GCC TAA GAT GAG CGC AAG TTG-3′, Mouse REV 5′-TAC TAG GCA GAT GGC CAC AGG ATT TT-3′; Rat FW 5′-GGT CCA TTC CTA TGA CTG TAG AAT TT-3′, Rat REV 5′-CCA TCA AGA CGT TCT CAG TT-3′.

### Western Blotting

The isolated left ventricle was snap-frozen, finely minced, and lysed using a buffer composed of 50 mM Tris-HCl, 150 mM NaCl, 10 mM *N*-ethyl maleimide, 1 mM EGTA, and 1% Triton X-100 (pH 7.5) (10), supplemented with protease inhibitors PMSF, leupeptin, and aprotinin. The resulting cell lysates were sonicated for 5 min, followed by centrifugation at 3,000 *g* for 15 min at 4°C. The supernatant was transferred to a fresh tube and subjected to a second centrifugation at 50,000 *g* for 45 min at 4°C. The soluble fraction was collected, and the protein concentration was determined using the Pierce BCA protein assay (ThermoFisher, 23222) with CuSO_4_. Sample lysates were diluted in a 4:1 ratio with sample buffer (60 mM Tris, pH 6.8, 14.4 mM β-mercaptoethanol, 25% glycerol, 2% SDS, and 0.1% bromophenol blue) and heated at 45°C for 10 min. Western blot analysis was also conducted on isolated ventricular cardiomyocytes. Here, cells were cultured in 6 cm petri dishes and incubated under standard conditions. Cells were washed with ice-cold phosphate-buffered saline (PBS) and lysed in 250 μL of lysis buffer containing 1% Triton X-100. A cell scraper was used to promote efficient lysis and detachment from the dish. Lysates were transferred to prechilled 1.5 mL tubes and sonicated in a water bath for 2 min. Subsequent centrifugation and protein determination/preparation steps were performed, as previously described. For protein separation, a pre-cast 1-mm-thick Mini-PROTEAN TGX 4%–15% gel (BioRad) with a sample volume of 15 μL/well in a 15-well format was used. Each lane was loaded with 25 μg of total protein. Electrophoresis was conducted at 70 V for 10 min, followed by 55 min at 200 V (33). Proteins were subsequently transferred onto a nitrocellulose membrane using a Mini Trans-Blot electrophoretic transfer cell (BioRad) via wet transfer at a constant 100 V for 3 h, with the chamber maintained in an ice bath (33). The transfer buffer consisted of 25 mM Tris, 192 mM glycine, and 20% methanol. Protein transfer was validated through Ponceau and Coomassie blue staining. Membranes were blocked with 5% skim milk in Tris-buffered saline containing 0.1% Tween-20 and 1 mM EDTA (py-TBST). Na_v_1.5 detection was performed using a rabbit monoclonal anti-Na_v_1.5 antibody (Cell Signaling, 14421) diluted 1:1,000 in py-TBST without BSA. Vinculin, used as a loading control, was detected using a rabbit monoclonal anti-vinculin antibody (Cell Signaling, E1E9V, 13901) diluted 1:1,000 in py-TBST containing 5% BSA, following the manufacturer’s recommendations. Membranes were incubated with primary antibodies at 4°C for 24 h under gentle agitation, then washed three times with py-TBST (5 min each). Subsequently, membranes were incubated for 1 h with a horseradish peroxidase-conjugated anti-rabbit secondary antibody (Cell Signaling, 7074S). After three additional 5-min washes with py-TBST and a final rinse with 1× Tris-buffered saline (TBS) (without TBS-T or EDTA), protein detection was performed using Pierce ECL Plus Western blotting substrate (ThermoFisher), and visualization was carried out using the ChemiDoc imaging system (BioRad). The Western blots were quantified with Image Studio (2023) with the integrated analysis tool, where the region of interest was drawn around the bands of each lane. The software then subtracted the background and gave a value of the intensity of the band compared to the background, which was then normalized to the housekeeper Vinculin and then to the wt. Six “biological” replicates per sample in “single” technical replicates were run. Full Western blot gel images are shown in Supplemental Fig. S2.

### Statistical Data Analyses

For all patch clamp data, comparisons were made using a nested analysis respecting the hierarchical data structure (measurements of *n* cells from *m* animals) detailed in Sikkel et al. (34). For TN-C incubation experiments, hierarchical testing was paired because cardiomyocytes incubated under the various conditions always originated from the same animals. For statistical tests used to analyze RT-qPCR and Western blot data, see respective figure legends. The tests were chosen based on the respective distribution of the data (normally distributed or not). A *P* value <0.05 was considered significantly different.

## Results

**Figure 1:**
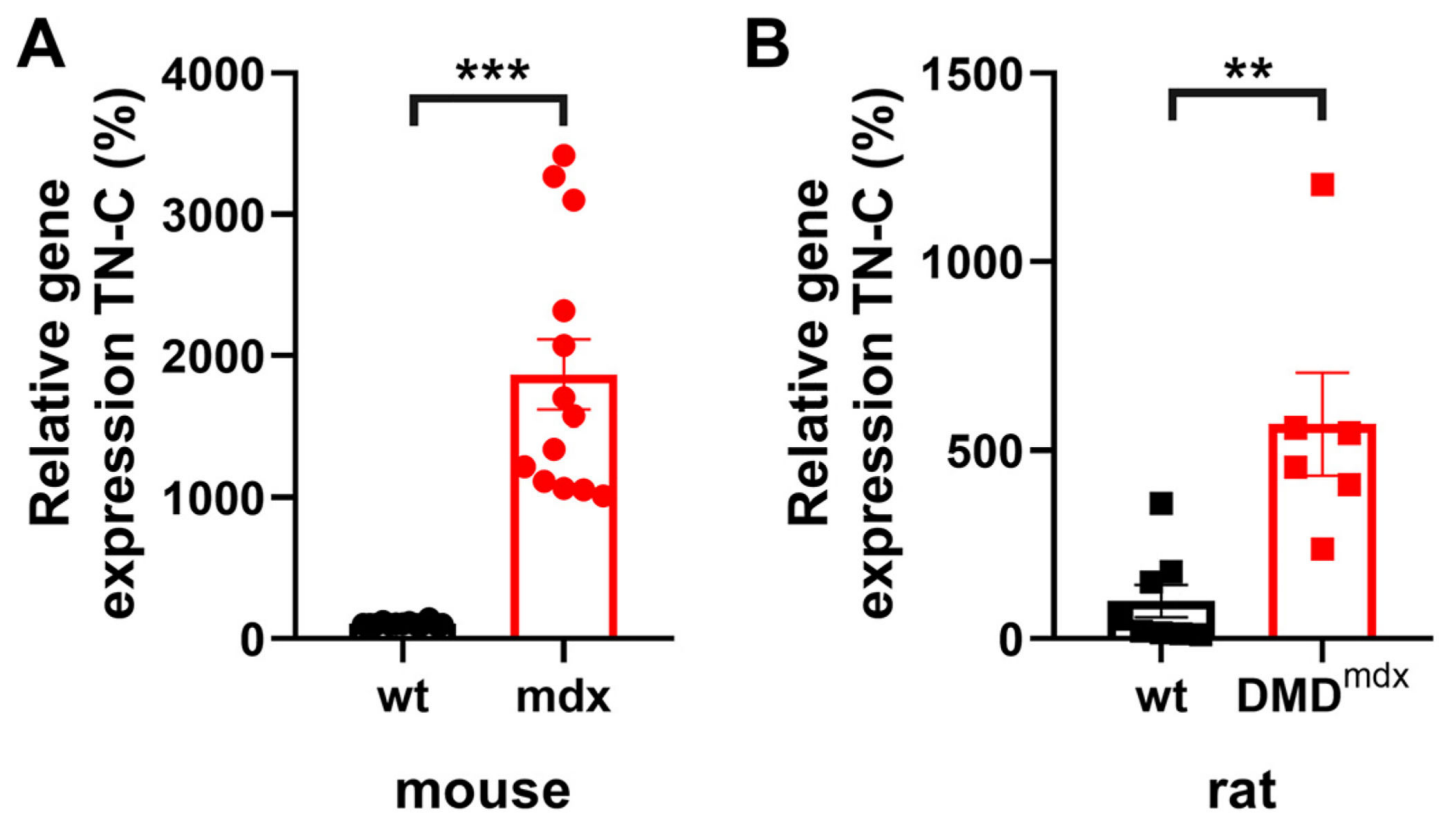
Gene expression levels of TN-C in cardiac tissues from adult male wt and dystrophic mice and rats. *A*: comparison of TN-C expression in left ventricular tissue from wt and *mdx* mice. *B*: TN-C expression in wt and *DMD*^mdx^ rat whole hearts. TN-C mRNA expression levels were normalized to the housekeeper HPRT1, and then to the mean relative expression of the wt (control group). Data are expressed as means ± SE [mice: *n =* 13 isolated hearts (one left ventricular tissue piece per heart) for both wt and *mdx*; rats: *n =* 8 isolated hearts for wt, *n =* 6 isolated hearts for DMD^mdx^]. Each data point represents the mean of two technical replicates of one biological sample. Statistical analyses were performed with a Mann–Whitney *U* test (unpaired). ^**^P < 0.01; ^***^P < 0.001. DMD, Duchenne muscular dystrophy; TN-C, tenascin C; wt, wild type.

Serum TN-C levels are significantly increased in patients with DMD (22) and *mdx* mice (23). Enhanced TN-C protein levels are also present in ventricular tissue from dystrophic mice and rats (24, 25). In the present study, increased expression of TN-C in mouse (Fig. 1A) and rat (Fig. 1B) dystrophic compared with normal healthy (wild-type, wt) cardiac tissue was confirmed by RT-PCR.

**Figure 2:**
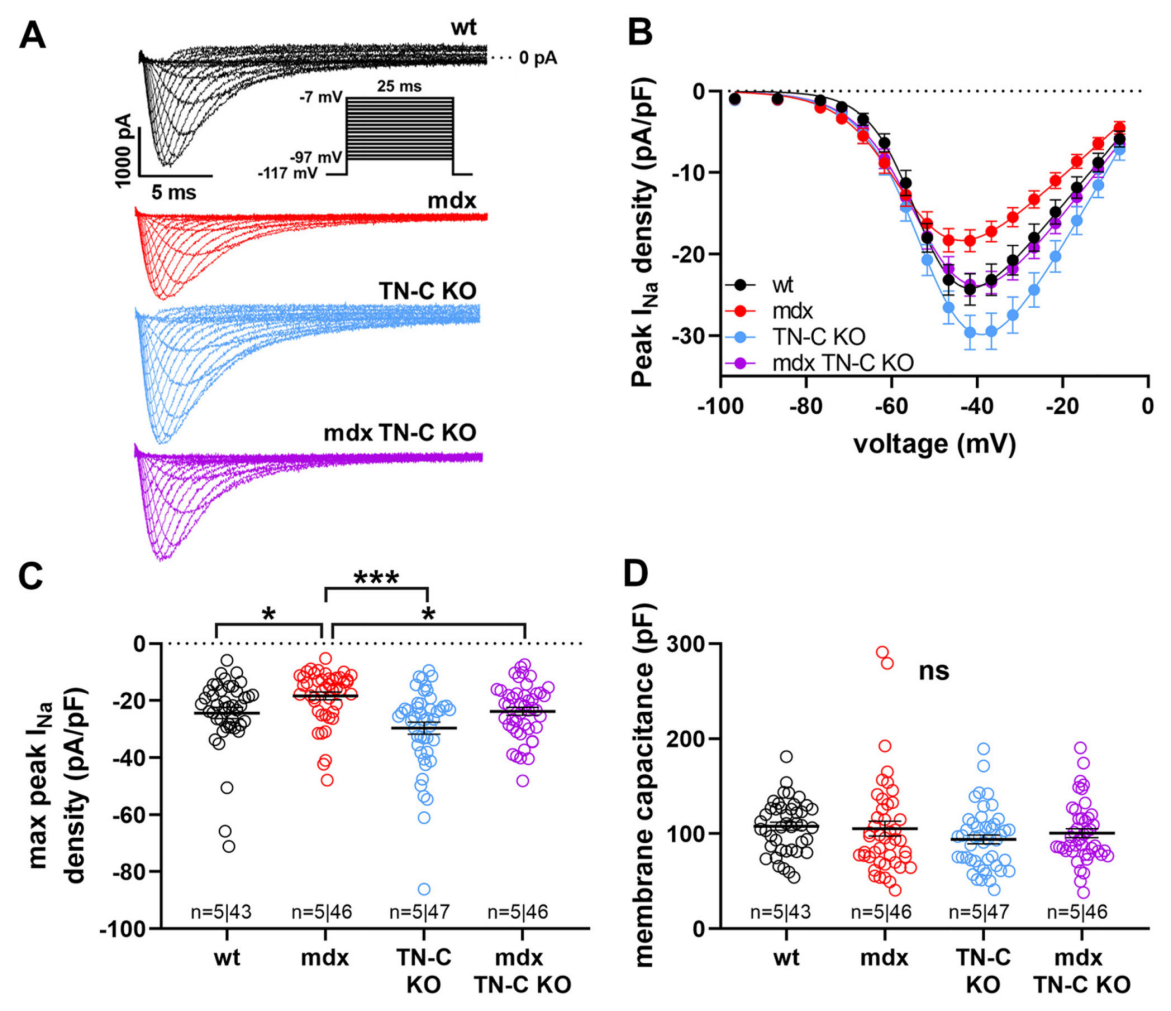
Comparison of the peak *I*_Na_ densities of ventricular cardiomyocytes derived from wt, dystrophin-deficient (*mdx*), TN-C KO, and *mdx*-TN-C double KO mice. *A*: representative original *I*_Na_ traces, recorded from myocytes of the different mouse lines, elicited by the pulse protocol shown in the *inset. B*: peak *I*_Na_ density-voltage relationships for wt, *mdx*, TN-C KO, and *mdx*-TN-C double KO myocytes. V_50_, the voltage at which half-maximum activation occurred, was −56 ± 1 mV for wt, −52 ± 2 mV for *mdx*, −56 ± 2 mV for TN-C KO, and −55 ± 1 mV for *mdx*-TN-C double KO myocytes (no significant difference between groups, *P* always > 0.24). *C*: comparison of the peak *I*_Na_ densities of myocytes from the different mouse lines at current maximum (−42 mV). ^*^P < 0.05; ^***^P < 0.001. wt vs. mdx: significantly different (*P* < 0.05) from −47 to −32 mV; mdx vs. mdx TN-C KO: from −42 to −32 mV; wt vs. TN-C KO: from −37 to −27 mV. *n* numbers represents the number of hearts (animals) used per group; the total number of cells is also given. *D*: comparison of the membrane capacitance values of the recorded myocytes as measure of cell size; ns, not significant. Data are expressed as means ± SE. *I*_Na_, sodium current; KO, knockout; TN-C, tenascin C; wt, wild type.

To test whether TN-C affected peak *I*_Na_ in wt and dystrophic hearts, we first recorded *I*_Na_ from ventricular cardiomyocytes derived from age-matched adult wt, *mdx*, TN-C KO, and *mdx*-TN-C double KO mice. Figure 2 shows that, in line with our earlier studies (6–8), the peak *I*_Na_ density in dystrophic *mdx* myocytes was significantly reduced when compared with the peak *I*_Na_ density in wt myocytes. This peak *I*_Na_ loss in *mdx* cardiomyocytes was fully rescued by additional knockout of TN-C (compare *I*_Na_ densities of wt, *mdx*, and *mdx*-TN-C KO cardiomyocytes; Fig. 2, B and C). Further, peak *I*_Na_ in myocytes derived from TN-C KO mice was increased by tendency (*P =* 0.09 at −42 mV) compared with wt. These findings suggested that a lack of TN-C expression in the heart induces Na channel gain-of-function both in healthy (wt) and dystrophic (*mdx*) cardiomyocytes. In addition, Na channel loss-of-function in dystrophin-deficient ventricular cardiomyocytes can be fully rescued by knockout of TN-C expression.

**Figure 3:**
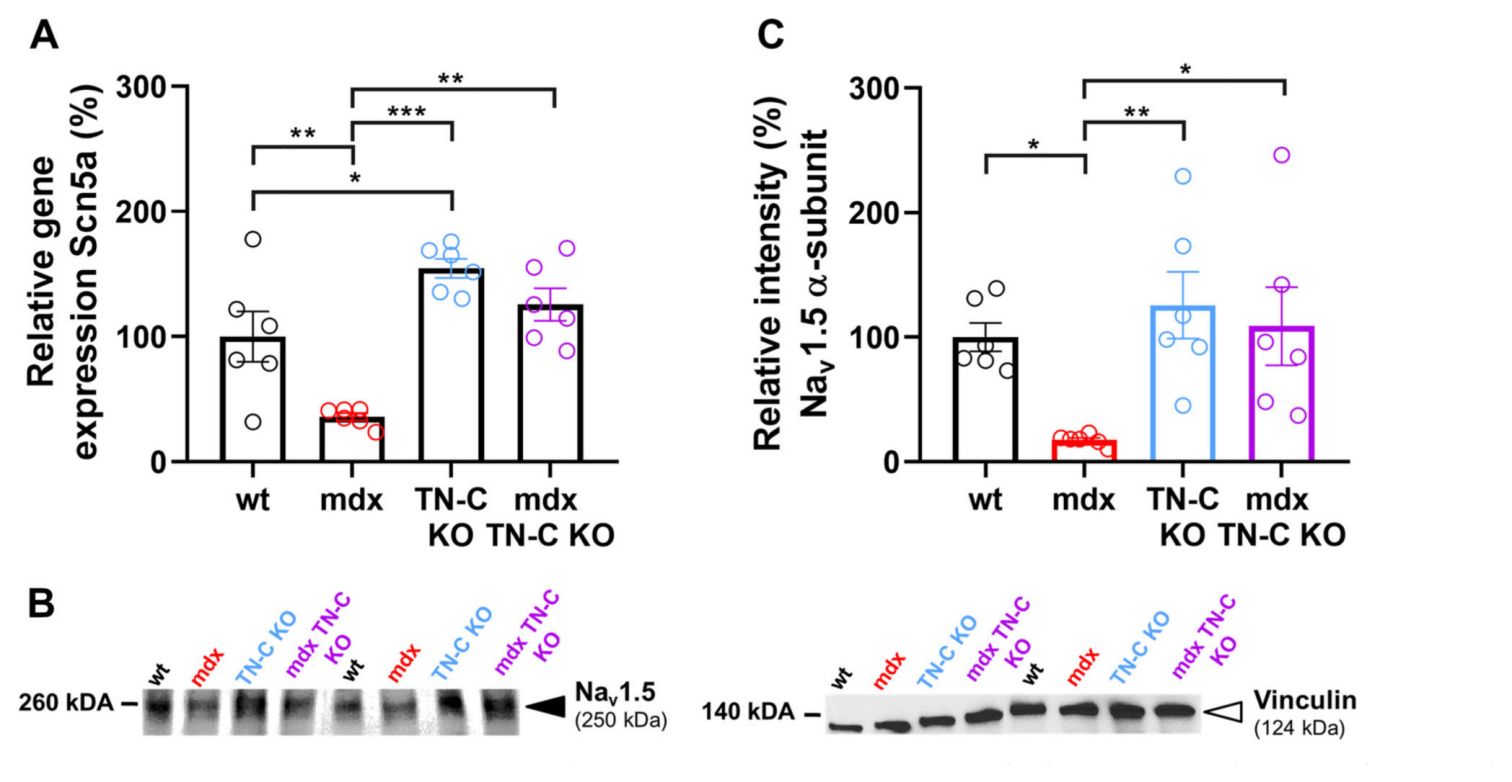
Cardiac Na channel gene and protein expression in left ventricular tissue from wt, *mdx*, TN-C KO, and *mdx*-TN-C double KO mice. *A: Scn5a* mRNA levels were normalized to the housekeeper HPRT1, and then to the mean relative expression of the wt (control group). Data are expressed as means ± SE. One way ANOVA revealed a significant difference between the four groups (*P* < 0.001). Post hoc comparison between two groups was performed with Holm–Sidak’s multiple comparisons test; ^*^P < 0.05, ^**^P < 0.01, ^***^P < 0.001. *n =* 6 hearts (one left ventricular tissue piece per heart) per genotype. Each data point represents the mean of two technical replicates of one biological sample (one isolated left ventricle). *B*: representative Western blot of left ventricular tissue. Black arrowhead: Na_v_1.5 α-subunit; white arrowhead: vinculin (loading control). *C*: densitometric quantification of Na_v_1.5 intensities normalized to the respective band intensities of vinculin, and then to the mean relative intensity for the wt. *n =* 6 hearts (one left ventricular tissue per heart) per genotype. Each data point represents one isolated heart. A Kruskal–Wallis test (unpaired) revealed a significant difference between the four groups (*P* < 0.01). Post hoc comparison between two groups was performed with Dunn’s multiple comparisons test; ^*^P < 0.05, ^**^P < 0.01. KO, knockout; TN-C, tenascin C; wt, wild type.

Na channel gain-of-function or loss-of-function in the absence or presence of TN-C, respectively, may be explained by modulation of Na channel expression by the protein. This was tested in RT-PCR and Western blot experiments performed on left ventricular tissue derived from wt, *mdx*, TN-C KO, and *mdx*-TN-C double KO mice. Figure 3 shows that Na_v_1.5 expression at mRNA (Fig. 3A) and protein (Fig. 3, B and C) level was significantly decreased in *mdx* compared with wt tissue. Na_v_1.5 expression in TN-C-KO tissue was significantly (mRNA level) and tendentially (protein level) increased compared with wt, and loss of Na_v_1.5 expression in *mdx* tissue (at both mRNA and protein level) compared with wt was fully rescued by additional knockout of TN-C. Together with the patch clamp data described earlier (Fig. 2), these results suggested that the presence of TN-C diminishes Na_v_1.5 channel expression, which results in reduced *I*_Na_ through the cardiomyocyte plasma membrane. Supplemental Fig. S3 shows that the Na channel β-1 subunit protein expression was significantly enhanced in *mdx* compared with wt left ventricular tissue. Probably, the strong downregulation of Na_v_1.5 α subunit expression in the absence of dystrophin may have led to a counterreaction, namely, upregulation of b-1 sub-unit expression, to limit Na_v_1.5 α subunit loss. Additional TN-C KO in *mdx* seemed to partly restore normal β-1 subunit expression, as no significant difference existed between wt and *mdx*-TN-C KO (Supplemental Fig. S3A). Na channel β-2 subunit expression was similar in all genotypes (Supplemental Fig. S3B).

**Figure 4:**
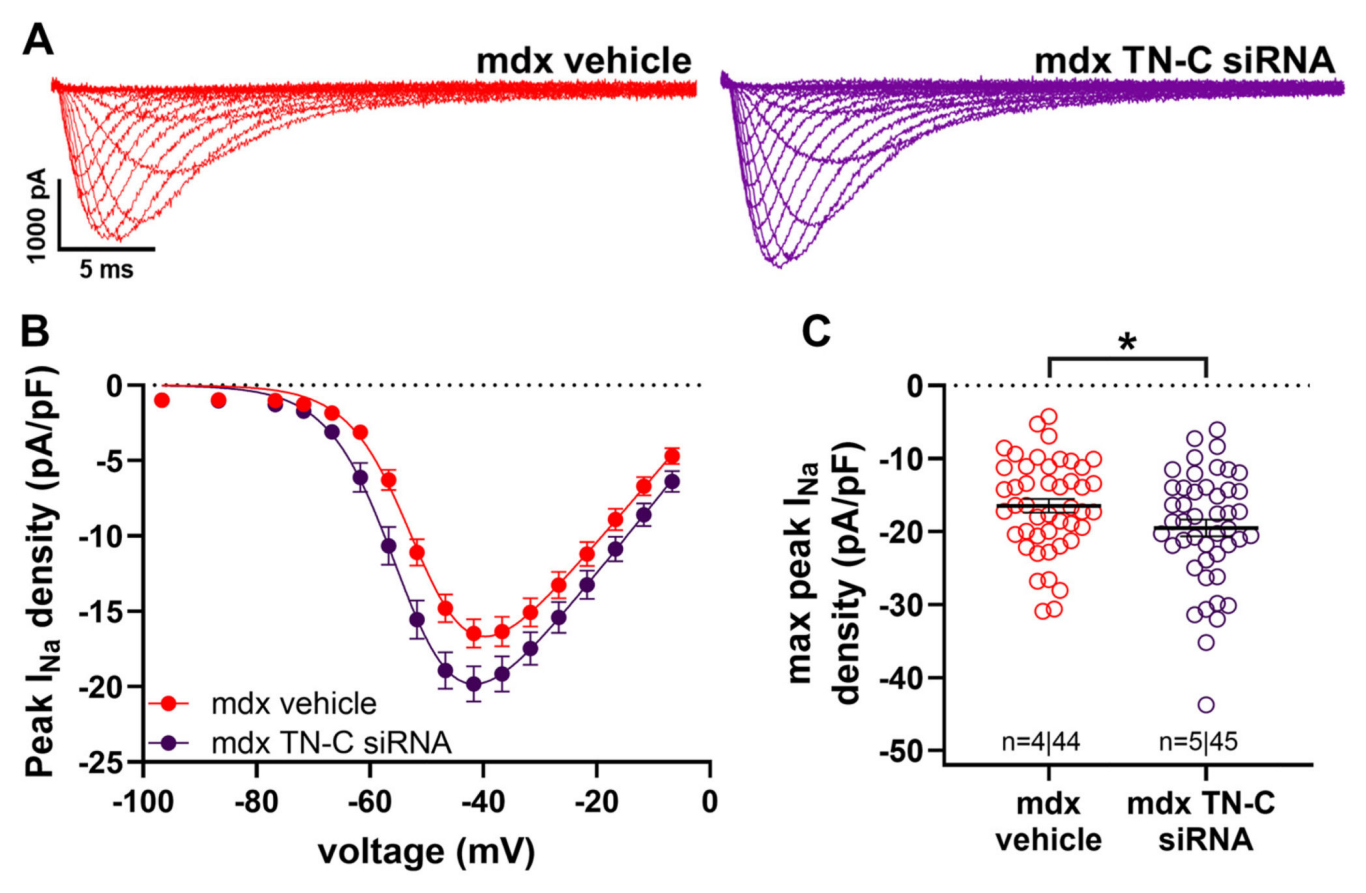
Effect of TN-C siRNA treatment of *mdx* mice on the peak *I*_Na_ densities of ventricular cardiomyocytes. *A*: original traces of *I*_Na_ recorded from myocytes derived from a vehicle (PBS)-(*left*) or siRNA-treated (*right*) *mdx* mouse. *B*: comparison of the peak *I*_Na_ density-voltage relationships of *mdx* myocytes derived from vehicle (PBS)- or siRNA-treated *mdx* mice. *C*: respective peak *I*_Na_ density comparison at current maximum (−42 mV). Data are expressed as means ± SE. ^*^P < 0.05. mdx vehicle vs. mdx TN-C siRNA: significantly different (*P* < 0.05) from 62 to −37 mV. *n* numbers represents the number of hearts (animals) used per group; the total number of cells is also given. The membrane capacitance values of myocytes from vehicle- and siRNA-treated mice were similar (*P =* 0.33). *I*_Na_, sodium current; TN-C, tenascin C.

Although TN-C KO mice develop a grossly normal phenotype and regular heart morphogenesis (17, 27), lack of TN-C during heart development may impact on Na channel expression/function in the adult heart. In an attempt to eliminate potential developmental effects, we carried out a series of experiments at which TN-C expression in *mdx* mice was knocked down with siRNA. Figure 4 shows that TN-C knockdown significantly increased the peak *I*_Na_ density in *mdx* cardiomyocytes. This suggested that, also in mice not lacking TN-C during development, TN-C inhibition induces Na channel gain-of-function in dystrophic (*mdx*) cardiomyocytes.

**Figure 5:**
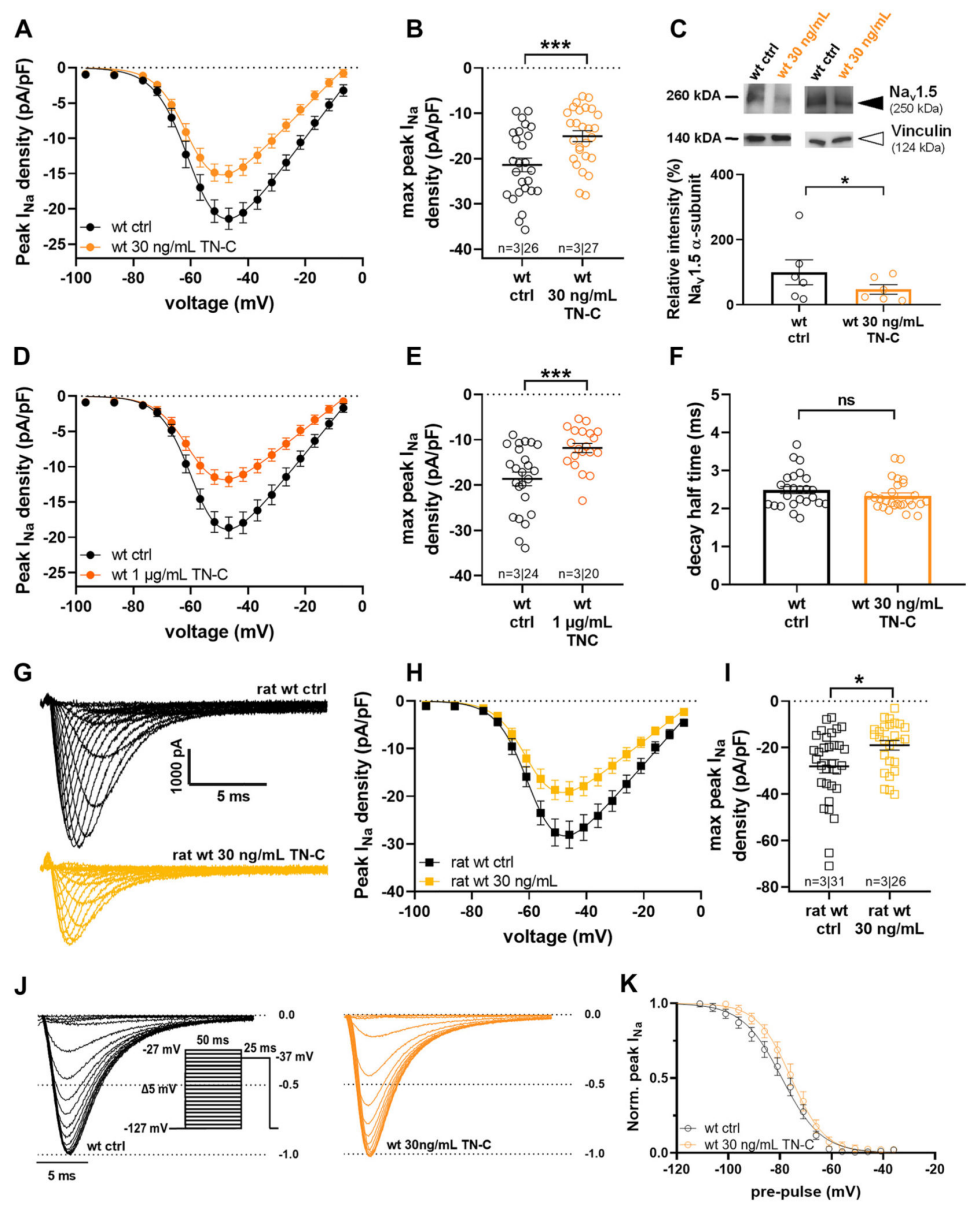
Effects of 24 h incubation of wild-type (wt) ventricular cardiomyocytes with human recombinant tenascin-C (hTN-C). *A*: peak *I*_Na_ density-voltage relationships of untreated and hTN-C (30 ng/mL)-treated wt myocytes. *B*: respective comparison of the peak *I*_Na_ densities at current maximum (−47 mV); ***P < 0.001. wt ctrl vs. wt 30 ng/mL TN-C: significantly different (*P* < 0.05) from −62 to −7 mV. *C*: representative Western blot (*top*) performed on isolated untreated and hTN-C (30 ng/mL)-treated wt ventricular cardiomyocytes. Black arrowhead: Na_v_1.5 α-subunit; white arrowhead: vinculin (loading control). Densitometric quantification of Na_v_1.5 α-subunit protein expression (membrane fraction) relative to vinculin (*bottom*), and then normalized to the mean relative intensity for the wt (*n* = 6 hearts for cardiomyocyte isolations; cells from each heart were plated onto two dishes: untreated and treated). A Wilcoxon matched-pairs signed rank test revealed a significant difference; *P < 0.05. *D*: peak *I*_Na_ density-voltage relationships of untreated and hTN-C (1 lg/mL)-treated wt myocytes. *E*: respective comparison of the peak *I*_Na_ densities at current maximum (− 47 mV); ^***^P < 0.001. wt ctrl vs. wt 1 lg/mL TN-C: significantly different (*P* < 0.05) from −57 to −12 mV. *F*: comparison of the kinetics of fast inactivation (represented by decay half-times) at −47 mV between untreated wt control (ctrl) and hTN-C (30 ng/mL)-treated wt myocytes. ns, not significant. *G*: representative *I*_Na_ traces from an untreated control (ctrl) wt rat ventricular cardiomyocyte and from a 24 h hTN-C (30 ng/mL)-treated wt rat ventricular cardiomyocyte. *H*: peak I_Na_ density-voltage relationships of untreated and hTN-C (30 ng/mL)-treated wt rat myocytes. *I*: respective comparison of the peak *I*_Na_ densities at current maximum (− 47 mV); *P < 0.05. Rat wt ctrl vs. rat wt 30 ng/mL TN-C: significantly different (*P* < 0.05) from −57 to −7 mV. The cardiomyocytes originated from 10 to 11-wk-old wt Sprague–Dawley rats. *J*: original I_Na_ traces from an untreated control (ctrl), and a 24 h hTN-C (30 ng/mL)-treated wt mouse ventricular cardiomyocyte elicited by a 25 ms test pulse to −37 mV. The pulse protocol designed to test for steady-state fast inactivation is displayed in the inset. *K*: comparison of the voltage dependencies of steady-state fast inactivation between untreated (ctrl) and hTN-C (30 ng/mL)-treated wt mouse ventricular cardiomyocytes. *V*_50_, the voltage at which half-maximum inactivation occurred, was -79 ± 2 mV for ctrl and −76 ± 2 mV for TN-C-treated myocytes and not significantly different (*P =* 0.16). In all experiments described in this figure, the membrane capacitance values of untreated and hTN-C-treated myocytes were similar (*P* always > 0.15). *I*_Na_, sodium current.

Having shown that TN-C knockout and/or inhibition leads to Na channel gain-of-function, we next tested the effects of 24-h incubation of isolated ventricular cardiomyocytes with human recombinant TN-C. Because peak *I*_Na_ in ventricular cardiomyocytes from TN-C KO compared with wt mice was increased (Fig. 2), we reasoned that the presence of TN-C would induce Na channel loss-of-function. Indeed, Fig. 5, A–E shows that TN-C at two different concentrations [physiological: 30 ng/mL (22, 31) and high: 1 μg/mL] in the culture medium significantly decreased the peak *I*_Na_ density in wt myocytes. In a separate set of experiments, action potentials were recorded from control and TN-C-treated (24 h, 30 ng/mL) wt cardiomyocytes. Supplemental Fig. S4, *A* and *B* show that TN-C treatment significantly reduced the action potential amplitude. This accorded with the reduced peak *I*_Na_ density in TN-C-treated myocytes. In addition, TN-C diminished the upstroke velocity of the action potential (Supplemental Fig. S4C). Due to the large data variability, however, this difference did just not reach statistical significance (*P =* 0.08). The action potential duration was not affected by TN-C (Supplemental Fig. S4D). In further accordance with the TN-C-induced diminution of peak *I*_Na_ density, 24-h incubation of wt cardiomyocytes with TN-C (30 ng/mL) decreased their Na_v_1.5 protein levels (Fig. 5C), suggesting a TN-C-induced reduction of Na_v_1.5 expression. The kinetics of fast inactivation, represented by decay half-times (at −47 mV), were similar in untreated control and TN-C-treated wt myocytes (Fig. 5F). Next, we tested whether a shorter TN-C incubation time than 24 h was sufficient to generate an effect on peak *I*_Na_. Supplemental Fig. S5 shows that already after 6 h incubation with TN-C, a significant decrease in peak *I*_Na_ of wt ventricular cardiomyocytes was present. This decrease was somewhat less pronounced when compared with 24 h incubation (compare with Fig. 5, A and B). Finally, Supplemental Fig. S6 suggests that, in contrast to TN-C-induced peak *I*_Na_ density reduction in wt cardiomyocytes, 24 h incubation with the protein did not affect peak *I*_Na_ density in myocytes derived from *mdx* mice.

To exclude that the observed inhibitory effect of TN-C on peak *I*_Na_ was a specific phenomenon only apparent in mouse ventricular cardiomyocytes, we performed an additional series of 24-h TN-C incubation experiments (30 ng/mL) with myocytes derived from 10 to 11-wk-old wt Sprague–Dawley rats. We found that TN-C decreased the peak *I*_Na_ density in rat myocytes to a similar extent as in mouse myocytes (Fig. 5, G–I).

Next, we tested whether TN-C impacts the voltage dependence of *I*_Na_ steady-state fast inactivation. Figure 5, J and K shows that 24-h incubation of wt myocytes with TN-C (30 ng/mL) did not considerably affect the voltage dependence of fast inactivation. Together with similar fast inactivation kinetics (Fig. 5F) and comparable voltage dependencies of activation (Fig. 5, A and D) in the presence of TN-C, this finding suggested that protein-induced alterations in channel gating properties do not play a significant role in the observed Na channel-loss-of-function.

**Figure 6:**
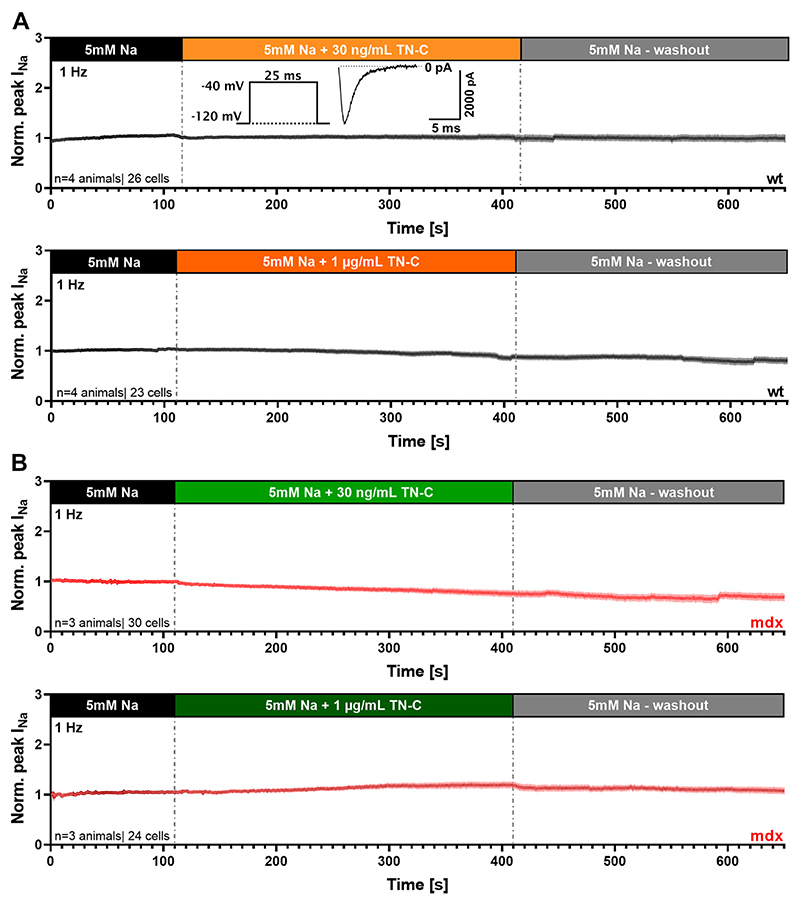
Acute application of TN-C to wt and *mdx* ventricular cardiomyocytes does not affect peak *I*_Na_. *A*: normalized peak *I*_Na_ of wt cardiomyocytes before, during superfusion with 30 ng/mL (*top*) or 1 μg/mL (*bottom*) hTN-C, and after washout. During the whole recording period *I*_Na_ was elicited by the pulse protocol shown in the inset at 1 Hz frequency. *n =* 26 cells from 4 wt hearts (30 ng/mL hTN-C) and 23 cells from 4 wt hearts (1 μg/mL hTN-C). *B*: respective experiment on *mdx* cardiomyocytes. *n =* 30 cells from 3 *mdx* hearts (30 ng/mL hTN-C) and 24 cells from 3 *mdx* hearts (1 μg/mL hTN-C). Data are expressed as means ± SE. *I*_Na_, sodium current; TN-C, tenascin C.

In a subsequent series of experiments, we tested whether TN-C exerts an immediate inhibitory effect on peak *I*_Na_ in ventricular cardiomyocytes, or if incubation with the protein (see Fig. 5) was needed to decrease the current amplitude. Figure 6 shows that acute superfusion of myocytes with bath solution containing TN-C (30 ng/mL or 1 lg/mL) during permanent *I*_Na_ recordings at 1 Hz stimulation frequency (for pulse protocol see *inset* in Fig. 6A) had no considerable effect on peak *I*_Na_. This was true for both wt (Fig. 6A) and *mdx* (Fig. 6B) cardiomyocytes, and control experiments on wt myocytes at 10 Hz stimulation frequency yielded a similar result (data not shown). These findings implied that peak *I*_Na_ reduction in ventricular cardiomyocytes by TN-C is not an acute effect, but requires incubation with or long-term presence of the protein.

**Figure 7:**
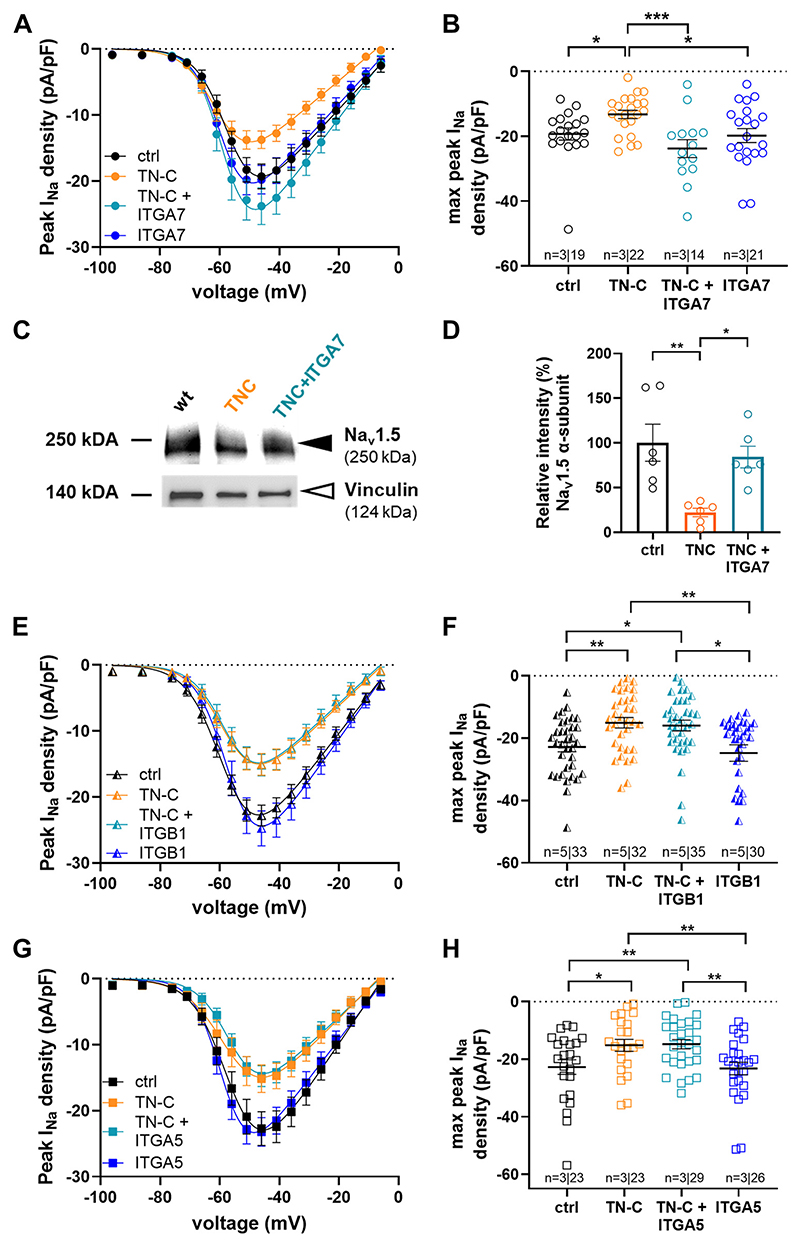
Effect of different integrin subunit blocking antibodies on the TN-C incubation-induced reduction in peak *I*_Na_ density. *A*: comparison of the peak *I*_Na_ density-voltage relationships of wt mouse ventricular cardiomyocytes either untreated (ctrl), 24 h hTN-C (30 ng/mL)-treated, 24 h hTN-C (30 ng/mL)- and integrin α-7 antibody (1:500)-treated, or only integrin α-7 antibody (1:500)-treated. *B*: respective peak *I*_Na_ density comparison at current maximum (−47 mV). ^*^P < 0.05, ^***^P < 0.001. ctrl vs. TN-C: significantly different (*P* < 0.05) from −52 to −12 mV; TN-C vs. TN-C ITGA7: significantly different (*P* < 0.05) from −57 to −12 mV. The membrane capacitance values of all groups were similar (*P* always > 0.26). *C*: Representative Western blot performed on isolated untreated, hTN-C (30 ng/mL)-treated, and hTN-C (30 ng/mL)-plus integrin α-7 antibody-treated wt ventricular cardiomyocytes. Black arrowhead: Na_v_1.5 α-subunit; white arrowhead: vinculin (loading control). *D*: densitometric quantification of Na_v_1.5 α-subunit protein expression (membrane fraction) relative to vinculin, and then normalized to the mean relative intensity for the wt control [*n =* 6 hearts for cardiomyocyte isolations; cells from each heart were plated onto three dishes: untreated, treated with TN-C, and treated with TN-C and integrin α-7 antibodies (1:500)]. A Kruskal–Wallis test (*P =* 0.0004) with Dunns’s multiple comparison revealed significant differences; *P < 0.05, **P < 0.01. *E*: comparison of the peak *I*_Na_ densityvoltage relationships of wt mouse ventricular cardiomyocytes either untreated (ctrl), 24 h hTN-C (30 ng/mL)-treated, 24 h hTN-C (30 ng/mL)- and integrin b-1 antibody (1:500)-treated, or only integrin b-1 antibody (1:500)-treated. *F*: respective peak *I*_Na_ density comparison at current maximum (−47 mV). *P < 0.05, **P < 0.01. ctrl vs. TN-C: significantly different (*P* < 0.05) from −72 to −7 mV; ctrl vs. TN-C ITGB1: significantly different (*P* < 0.05) from −72 to −7 mV. The membrane capacitance values of all groups were similar (*P* always > 0.71). *G*: comparison of the peak *I*_Na_ density-voltage relationships of wt mouse ventricular myocytes either untreated (ctrl), 24 h hTN-C (30 ng/mL)-treated, 24 h hTN-C (30 ng/mL)- and integrin α-5 antibody (1:500)-treated, or only integrin α-5 antibody (1:500)-treated. *H*: respective peak *I*_Na_ density comparison at current maximum (−47 mV). *P < 0.05, **P < 0.01. ctrl vs. TN-C: significantly different (*P* < 0.05) from −57 to −17 mV; ctrl vs. TN-C ITGA5: significantly different (*P* < 0.05) from −62 to −17 mV. The membrane capacitance values of all groups were similar (*P* always > 0.2). *I*_Na_, sodium current; TN-C, tenascin C.

TN-C effects on cardiomyocyte peak *I*_Na_ only after incubation, but not during acute superfusion with the protein (see Figs. 5 and 6), may suggest an indirect mechanism of action, possibly via binding of TN-C to surface receptors and subsequent intracellular signaling. In a final set of experiments, we tested whether binding of TN-C to α-7 integrin receptor subunits in the cardiomyocyte plasma membrane is necessary for modulation of peak *I*_Na_. Therefore, α-7 subunit blocking antibodies were used in 24-h TN-C incubation experiments. Figure 7, *A* and *B* show that the block of integrin α-7 subunits in wt cardiomyocytes during TN-C incubation prevented the normally observed reduction in peak *I*_Na_ induced by the presence of the protein. Accordingly, the TN-C-induced downregulation of Na_v_1.5 expression was impeded by blocking integrin α-7 subunits (Fig. 7, C and D). These findings suggested that TN-C may act via α-7 integrin binding and subsequent signaling. Finally, to check the specificity of α-7 integrin activity, we tested two other integrin subunits, *β*-1 and α-5, by using respective blocking antibodies. Figure 7 shows that, in contrast to integrin α-7 blockade (Fig. 7, A and B), neither blocking integrin *β*-1 (Fig. 7, E and F) nor integrin α-5 subunits (Fig. 7, G and H) during TN-C incubation prevented peak *I*_Na_ reduction induced by the presence of the protein.

## Discussion

**Figure 8:**
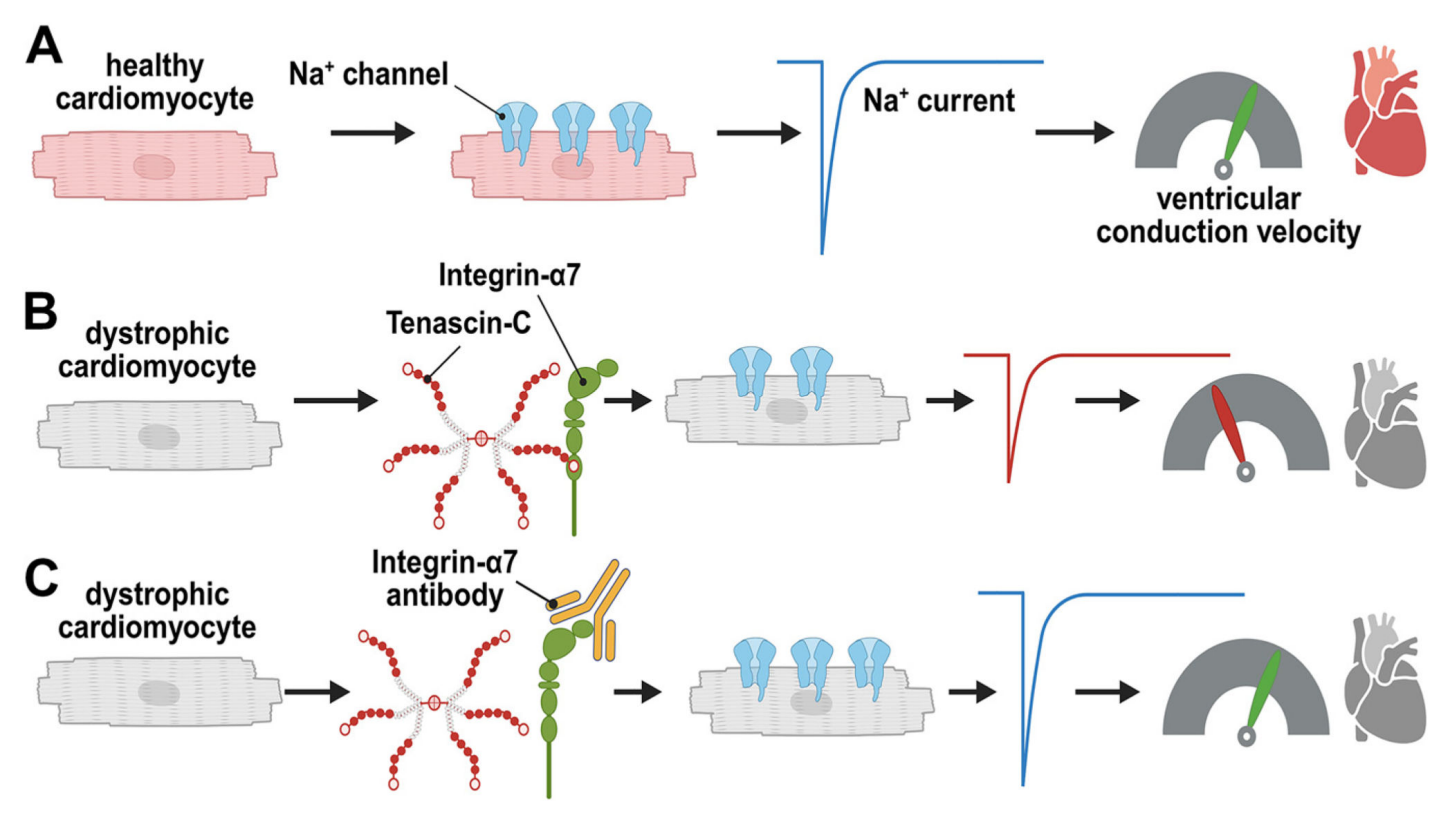
Summary of the findings of the present study and their potential impact. *A*: in healthy ventricular cardiomyocytes, a large number of Na channels are expressed in the plasma membrane, which give rise to a big *I*_Na_ and consequently a high ventricular conduction velocity. *B*: in dystrophic cardiomyocytes, TN-C interacts with integrin α-7, leading to reduced Na channel expression, diminished *I*_Na,_ and finally slowed ventricular conduction. *C*: by blocking integrin α-7 activity in dystrophic cardiomyocytes, normal Na channel expression, *I*_Na_, and ventricular conduction velocity are restored. Figure created with a licensed version of Biorender.com. *I*_Na_, sodium current; TN-C, tenascin C.

In the present paper, in accordance with the literature (24, 25), we report significantly enhanced expression of TN-C in the dystrophic heart. Ventricular cardiomyocytes from TN-C KO mice had increased peak *I*_Na_, and the abnormally reduced peak *I*_Na_ in dystrophin-deficient *mdx* myocytes was rescued to wt level by additional TN-C KO. This was accompanied by enhanced Na_v_1.5 channel expression in the absence of TN-C. Further, peak *I*_Na_ in dystrophic cardiomyocytes could be increased by treatment of *mdx* mice with TN-C siRNA. Twenty-four-hour incubation of wt myocytes with human recombinant TN-C reduced their peak *I*_Na_, an effect which could be abolished by blocking antibodies specific for the α-7 integrin subunit. Finally, acute application of TN-C had no effect on peak *I*_Na_ of wt and *mdx* cardiomyocytes. Together, these findings suggest that TN-C induces electrical remodeling in the dystrophic heart. Durable presence of this protein causes Na channel loss-of-function, and inhibition of its expression rescues abnormally reduced peak *I*_Na_ in dystrophic cardiomyocytes. The main findings and the potential impact of the present study are summarized in Fig. 8.

### Modulation of Peak *I*_Na_ in Ventricular Cardiomyocytes by TN-C

Via the dystrophin-associated protein complex (DAPC) dystrophin links the intracellular microfilament network of actin and the extracellular matrix (ECM) (35). Several ion channels (e.g., Na_v_1.5 channels) are DAPC members [reviewed in Koenig et al. (36)] and, as such prone to regulation by ECM proteins. Tenascins (TN-C and TN-R) were shown to directly interact with neuronal Na channels, affecting their localization and functional properties (37–39). In addition, tenascins interact with cell surface receptors such as integrins to induce signaling and affect gene expression in the heart (17, 40). Thereby, they may indirectly regulate ion channel expression and function.

Reduced peak *I*_Na_ in cardiomyocytes in the presence of TN-C may be explained by direct interaction of the protein with the Na channel, or by an indirect mechanism via receptor binding and subsequent signaling. Our data point to an indirect mechanism for two reasons. First, TN-C did not exert an immediate effect on peak *I*_Na_, but incubation with the protein was needed to decrease the current amplitude. Secondly, the application of blocking antibodies specific for the α-7 integrin subunit to cardiomyocytes abolished the effect of TN-C incubation on peak *I*_Na_, suggesting that TN-C acts via α-7 integrin binding and subsequent signaling. Alpha-7, the main integrin subunit detected in adult cardiomyocytes (41, 42), was indeed shown to bind TN-C (43–45). Future studies should prove whether TN-C/α-7 integrin signaling alters transcription via known pathways (e.g., integrin-linked kinase, ERK) to affect Na_v_1.5 expression. Irrespective of a direct or indirect mechanism of action, TN-C may reduce peak *I*_Na_ in ventricular cardiomyocytes by respective modulation of Na channel gating, or by diminishing functional Na channel expression in the plasma membrane. In our hands, TN-C neither considerably affected the voltage dependence of Na channel activation nor that of fast inactivation. The kinetics of fast inactivation were also independent of the presence of this protein. These findings suggested that the Na channel gating properties are not significantly affected by TN-C. In contrast, our Western blot experiments showed that Na channel protein levels in cardiomyocytes are considerably modulated by TN-C. Thus, absence of TN-C in dystrophic *mdx* cardiomyocytes led to increased Na_v_1.5 protein levels, which may explain peak *I*_Na_ enhancement. In contrast to Na_v_1.5 (alpha subunit) expression, the expression of Na channel *β*-1 and *β*-2 subunits was not considerably affected by the absence or presence of TN-C. This suggested that TN-C regulation of Na_v_1.5 expression occurs independent of Na channel beta subunits.

Taken together, our findings suggest that the durable presence of TN-C reduces functional Na channel expression in the cardiomyocyte plasma membrane. This occurs via an indirect mechanism that requires binding of TN-C to α-7 integrin subunits.

### Therapeutic Potential of TN-C Inhibition

Slowed ventricular impulse conduction represents a relevant source for cardiac arrhythmias in patients with DMD (3, 4). The present study suggests that this problem may partly be caused or at least aggravated by upregulation of TN-C in the dystrophic heart, which elicits electrical remodeling, i.e., *I*_Na_ loss-of-function in cardiomyocytes. Consequently, TN-C inhibition emerges as a strategy to counteract ventricular conduction impairments and arrhythmias in the dystrophic heart. Aimed inhibition of electrical remodeling by TN-C may also be beneficial for numerous other cardiac disease conditions in humans, in the course of which TN-C expression is significantly upregulated (14–17). Finally, TN-C inhibitory strategies may also be advantageous for the diseased heart via other mechanisms such as counteraction of the protein’s profibrotic and proinflammatory effects (46). In accordance, micro-dystrophin gene therapy in a mouse model of DMD prevented fibrosis-associated TN-C accumulation in heart tissue, concomitant with attenuated cardiac pathology and improved functionality (24). Finally, we want to point out that, besides harmful, TN-C may also exert protective effects in the course of cardiovascular diseases (e.g., Refs. 47, 48). Moreover, TN-C has a wide range of effects on non-cardiovascular tissues and modulates numerous physiological and pathophysiological processes. For example, the protein promotes tissue healing (48). Thus, systemic risks of TN-C inhibition need to be taken into account, and localized or tissue-specific TN-C inhibitory strategies should be applied.

### Study Limitations

From our data, it cannot definitely be concluded if TN-C has a similar or a different effect in the presence and in the absence of dystrophin. Figure 2, B and C suggest that peak *I*_Na_ density is increased by TN-C KO both in wt and *mdx* cardiomyocytes. However, a statistically significant difference (at −42 mV, current maximum, Fig. 2C) was only present in the absence of dystrophin (*mdx*). On the other hand, 24 h incubation of cardiomyocytes with TN-C reduced peak *I*_Na_ density only in wt (Fig. 5), but not in *mdx* myocytes (Supplemental Fig. S6). Probably, only wt cardiomyocytes, which are normally only exposed to very low concentrations of TN-C, react to TN-C incubation. *mdx* cardiomyocytes, on the other hand, are continuously exposed to considerable TN-C concentrations and may therefore not significantly react to TN-C incubation.

Further, we do not show protein expression data from ventricular tissue of TN-C siRNA- and vehicle-treated *mdx* mice, which would allow for comparison with the respective *I*_Na_ data. We also refrained from performing RT-qPCR and Western blot experiments on rat ventricular tissue (Fig. 5, G–I), because the rat studies only served control purposes for comparison with the mouse studies.

Moreover, whole hearts were used for rat qPCR, while left ventricular tissue for mouse qPCR (Fig. 1). For Western blot experiments, only left ventricular tissue was used, whereas whole ventricle was used for cell isolations to perform patch clamp studies. This may have generated a potential mismatch in the experimental comparisons (expression vs. function).

Only ventricular cardiomyocytes from the working myocardium (whole ventricle) were tested. Hence, we do not know if other cardiac cell types (e.g., Purkinje fibers and atrial myocytes) and human induced pluripotent stem cell-derived cardiomyocytes (from healthy individuals and DMD patients) are equally affected by TN-C. Further, differences may exist between right and left ventricular myocytes, as well as between epi- and endocardial myocytes. This may well have contributed to the data variability observed in the present study.

Finally, surface ECG recordings on the various mouse models used, and ventricular conduction velocity measurements (e.g., by optical mapping) have not been performed. Such approaches would provide essential functional validation and should be performed in the future.

## Supplementary Material

Supplementary Material

## Figures and Tables

**Figure:**
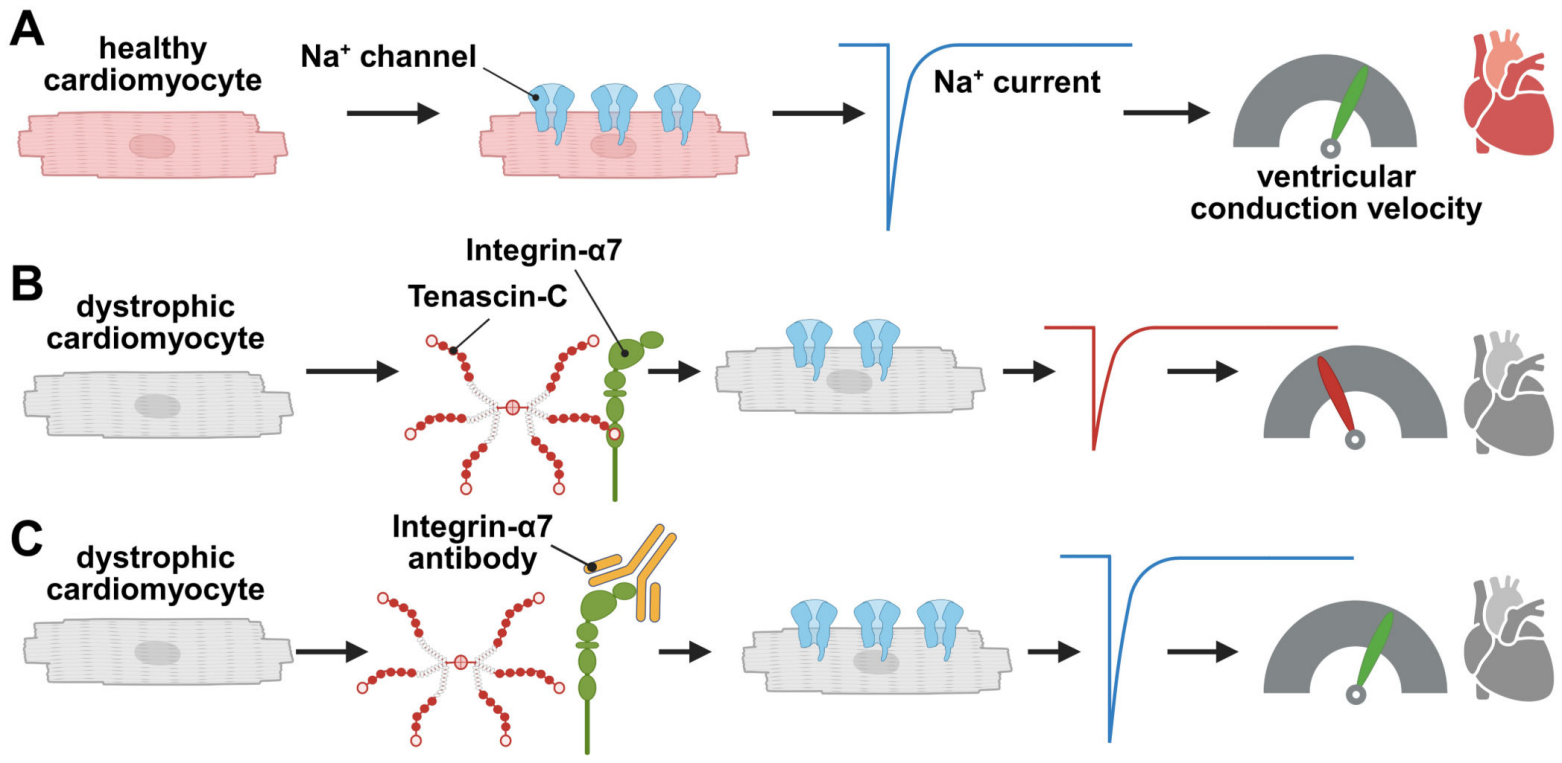


## Data Availability

The data underlying this article will be shared on reasonable request to the corresponding author.

## New & Noteworthy

Dystrophin deficiency in cardiomyocytes leads to abnormally reduced Na currents. These can be rescued by inhibition of the expression of tenascin C.
