# Supplementary material for: Inhibition of tenascin C rescues abnormally reduced Na currents in dystrophin-deficient ventricular cardiomyocytes
Source: Am J Physiol Heart Circ Physiol. Author manuscript; Available in PMC 2025 Sep 9. (PMC7618084; doi:10.1152/ajpheart.00307.2025)
Supplement: Supplementary Material [file EMS207941-supplement-Supplementary_Material.pdf]

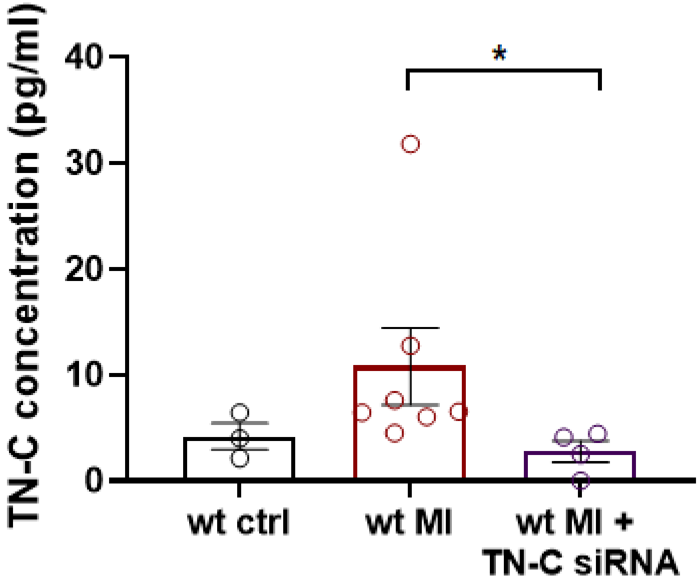

Supplementary Figure 1

Mouse plasma TN-C levels in wild-type (wt) mice with or without (ctrl) myocardial infarction (MI), and with or without TN-C siRNA knockdown treatment. Intraperitoneal (ip) injection of TN-C siRNA- (100 µg) on day 3 post myocardial infarction markedly reduced the expression of circulating (plasma) levels of TNC at day 7 in mice (n = 3-7 per group). Data are expressed as means ± SE. Serum was collected from mice by taking whole blood and centrifuged in Serum Sep Clot Activator blood tubes (Vacuette® tube) for 10 minutes at 4 °C, 3000 rpm. Then ELISA for TN-C was performed according to the protocol of the manufacturer. Mouse TN-C ELISA kit was purchased from Elabscience. A Kruskal-Wallis test revealed a significant difference between the 3 groups (P=0.004). Post hoc comparison between two groups was performed with Dunn's multiple comparisons test; *P<0.05.

Supplementary Figure 2 Full western blot gel images

ad **Figure 3**

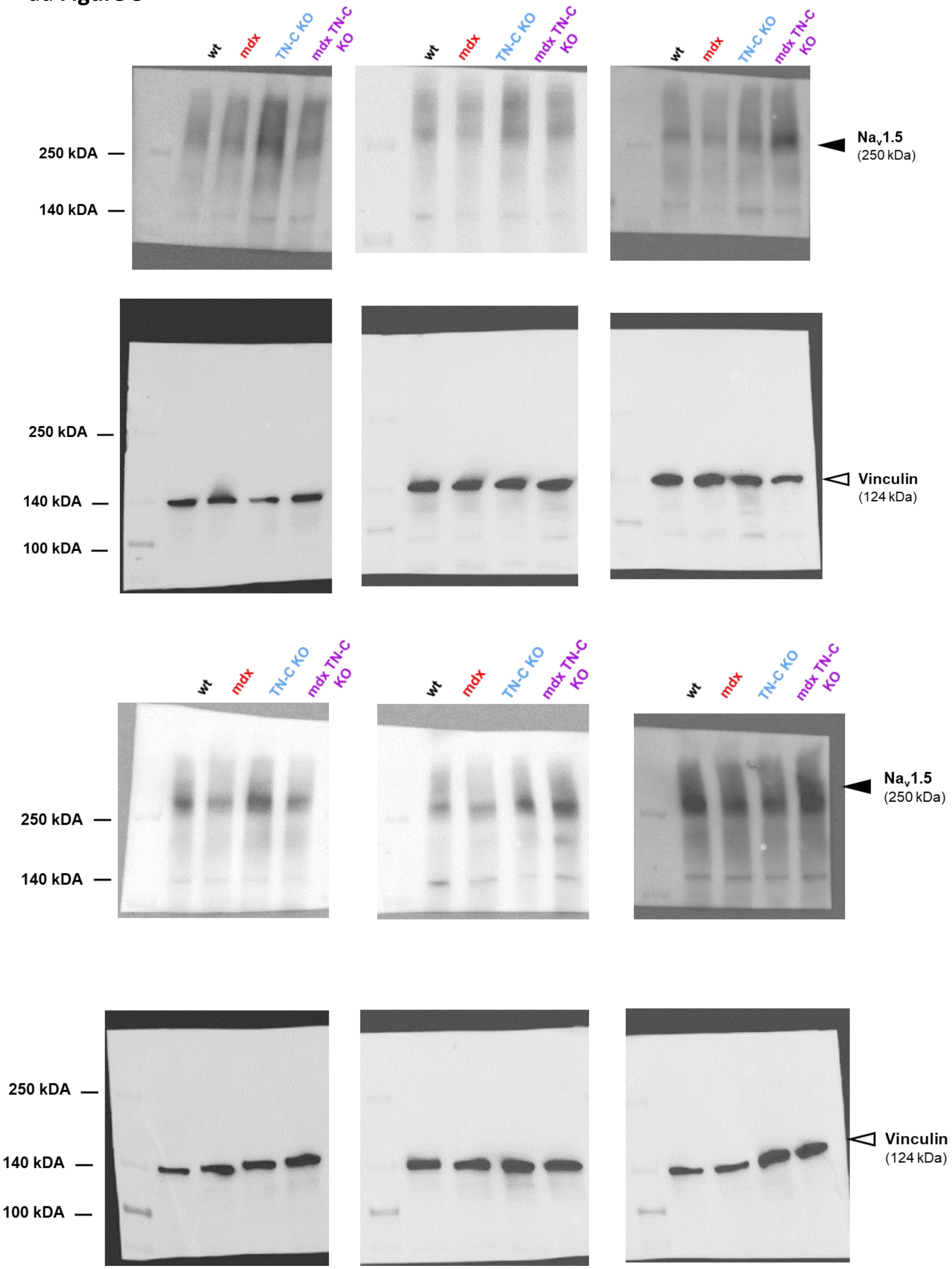

ad **Figure 5**

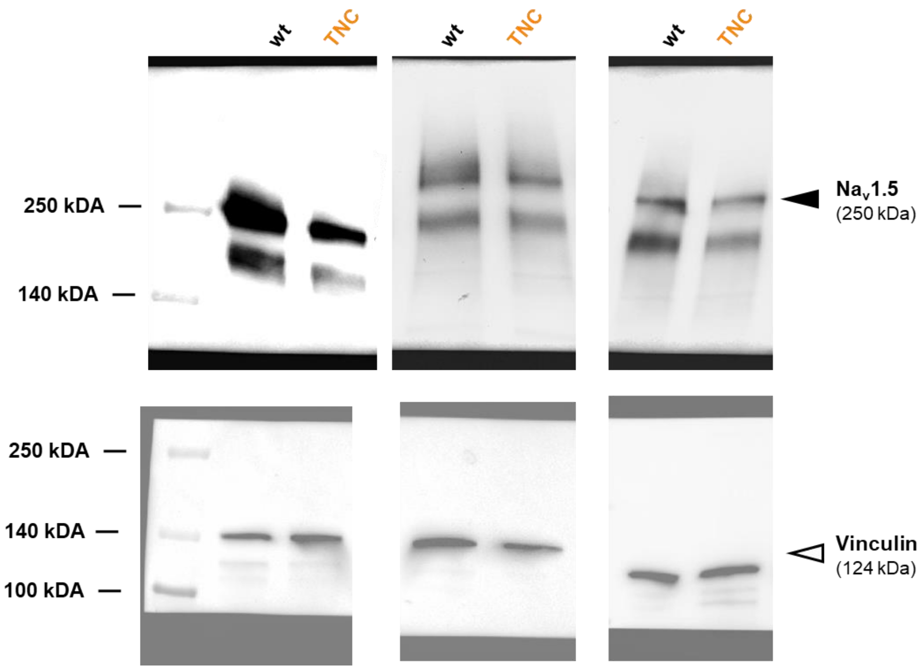

wt
TNC
wt
TNC
wt
TNC
250 kDA
140 kDA
Na$_v$1.5
(250 kDa)
250 kDA
140 kDA
100 kDA
Vinculin
(124 kDa)

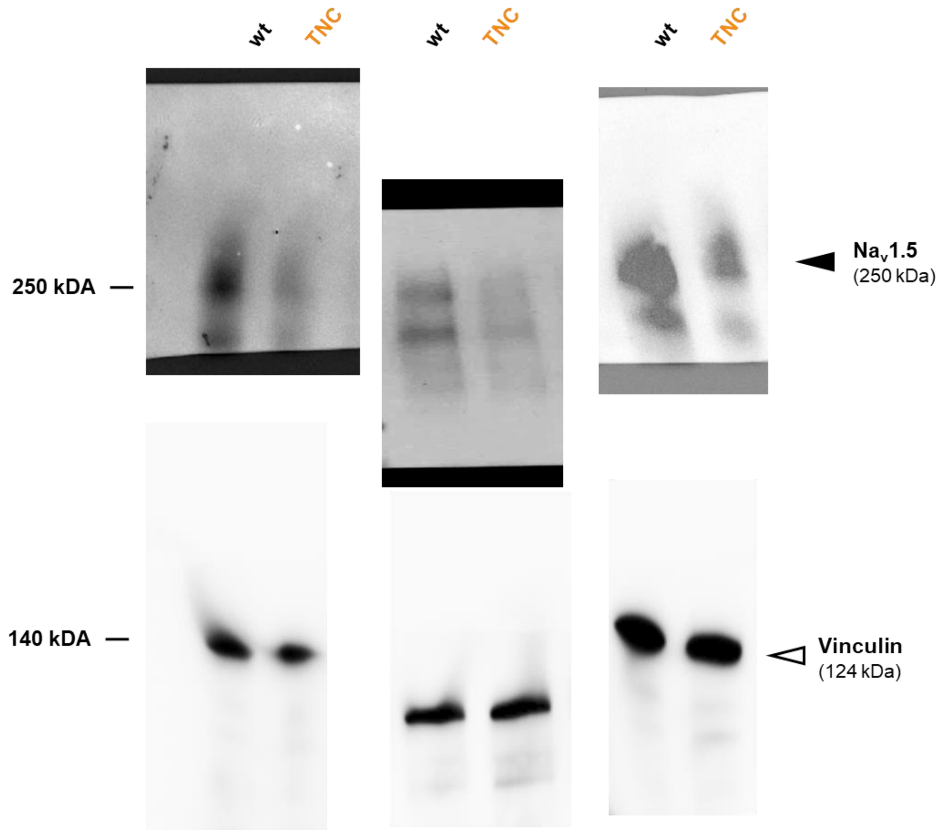

wt
TNC
wt
TNC
wt
TNC
250 kDA
Na$_v$1.5
(250 kDa)
140 kDA
Vinculin
(124 kDa)

ad **Figure 7**

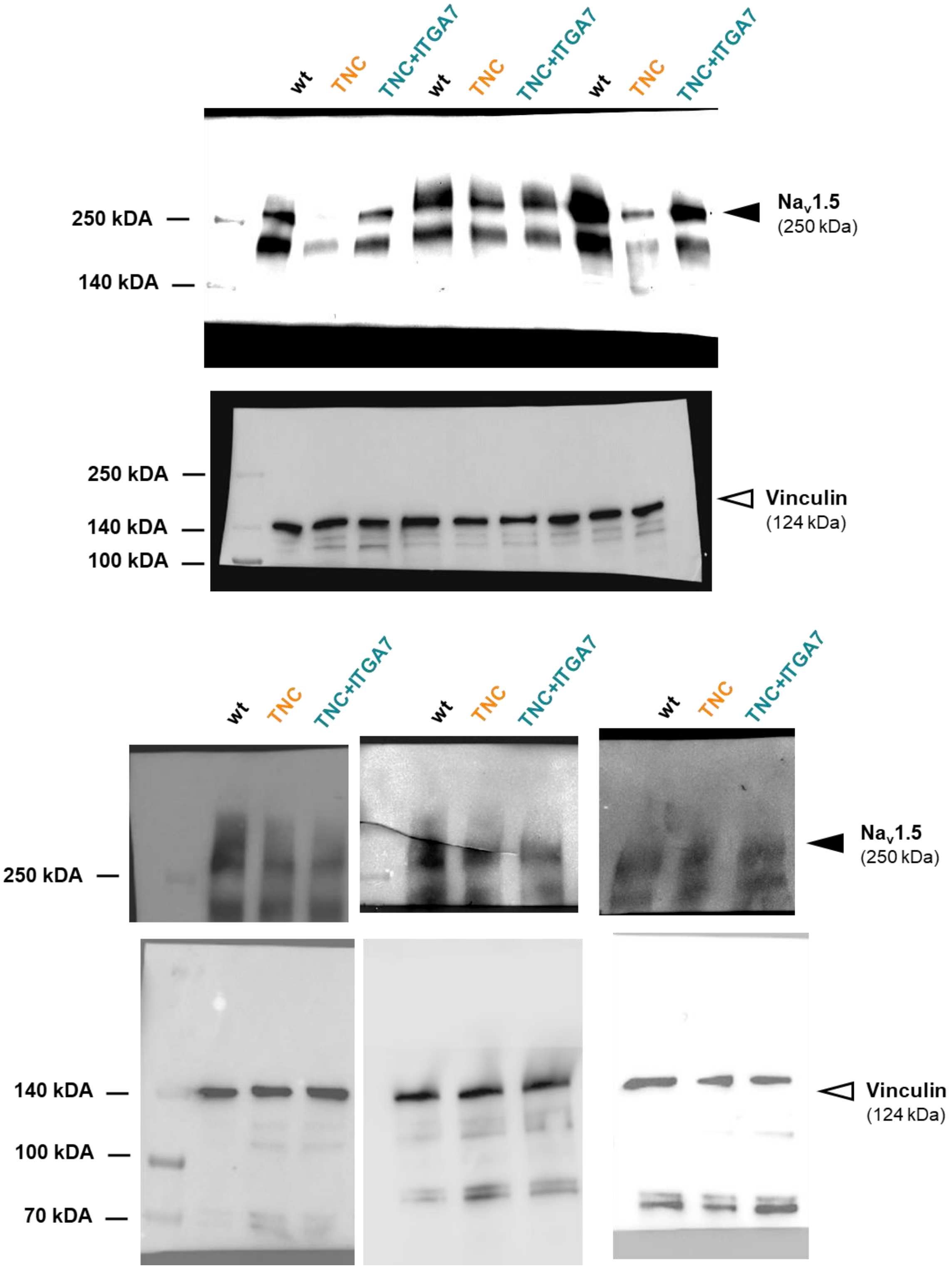
wt
TNC
TNC+ITGA7
250 kDA
140 kDA
100 kDA
70 kDA
$Na_v1.5$
(250 kDa)
Vinculin
(124 kDa)

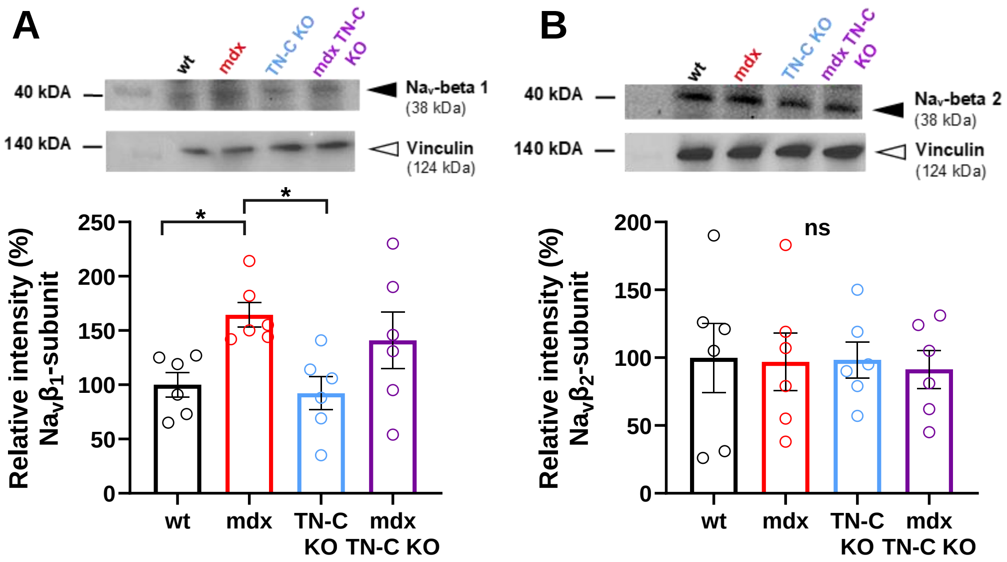

Supplementary Figure 3

Cardiac Na channel beta subunit protein expression in left ventricular tissue from wt, *mdx*, TN-C KO, and *mdx*-TN-C double KO mice. (A) Representative western blot for the Na channel beta-1 subunit of left ventricular tissue (top). Black arrowhead: Na channel beta-1 subunit; white arrowhead: vinculin (loading control). Na channel beta-1 subunit rabbit monoclonal antibody (cell signaling, D4Z2N, #13950). Densitometric quantification (bottom) of beta-1 subunit intensities normalized to the respective band intensities of vinculin, and then to the mean relative intensity for the wt. n = 6 hearts (one left ventricular tissue per heart) per genotype. Each data point represents one isolated heart. Data are expressed as means ± SE. A Kruskal-Wallis test revealed a significant difference between the 4 groups (P<0.05). Post hoc comparison between two groups was performed with Dunn's multiple comparisons test. *P<0.05. (B) Representative western blot for the Na channel beta-2 subunit of left ventricular tissue (top). Black arrowhead: Na channel beta-2 subunit; white arrowhead: vinculin (loading control). Na channel beta-2 subunit rabbit monoclonal antibody (cell signaling, D1S8E, #14686). Densitometric quantification (bottom) of beta-2 subunit intensities normalized to the respective band intensities of vinculin, and then to the mean relative intensity for the wt. n = 6 hearts (one left ventricular tissue per heart) per genotype. Each data point represents one isolated heart. ns, not significantly different. A Kruskal-Wallis test revealed no significant difference between the 4 groups (P>0.99); ns, not significantly different.

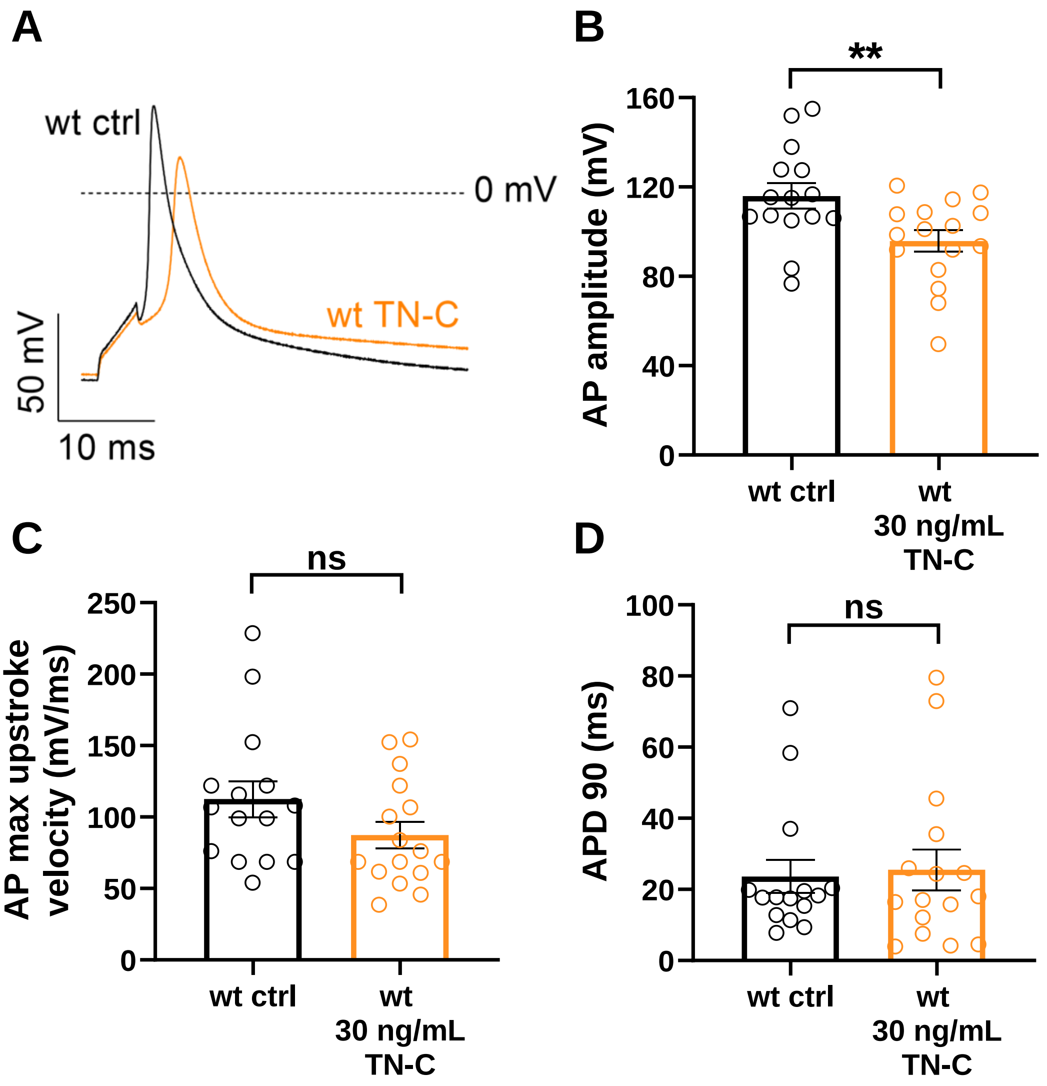

Supplementary Figure 4

Effects of 24 h incubation of wt mouse ventricular cardiomyocytes with human recombinant tenascin-C (hTN-C) on the action potential (AP). (A) Typical APs of ventricular cardiomyocytes from wt mice recorded in the current clamp mode of the whole cell patch clamp technique. The cardiomyocytes had been incubated for 24 h under control conditions (ctrl), or in the presence of 30 ng/mL TN-C. The resting membrane potential was fixed at approximately -85 mV by continuous current injections. Action potentials were elicited at a frequency of 10 Hz by rectangular current pulses of 4 ms duration at approximately 125 % threshold level. The cardiomyocytes were bathed in a solution containing (in mM) 140 NaCl, 4 KCl, 2 $CaCl_2$, 2 $MgCl_2$, 5 HEPES, 5 Glucose, and 0.017 blebbistatin, pH adjusted to 7.4 with NaOH. The pipette solution consisted of (in mM) 10 NaCl, 140 KCl, 2 EGTA, 1 $MgCl_2$, 0.1 Na-GTP, 5 Mg-ATP, and 10 HEPES, adjusted to pH 7.2 with KOH. The AP amplitude (B), the maximal upstroke velocity (C), and the AP duration at 90 % repolarization (APD 90) (D) are displayed (n = 15 cells for ctrl

and 16 cells for TN-C-treated derived from three wt mice). Statistical comparison was made using a nested analysis respecting the hierarchical data structure (measurements of n cells from m animals) detailed in Sikkel et al. (manuscript ref. 34). **P<0.05; ns, not significantly different.

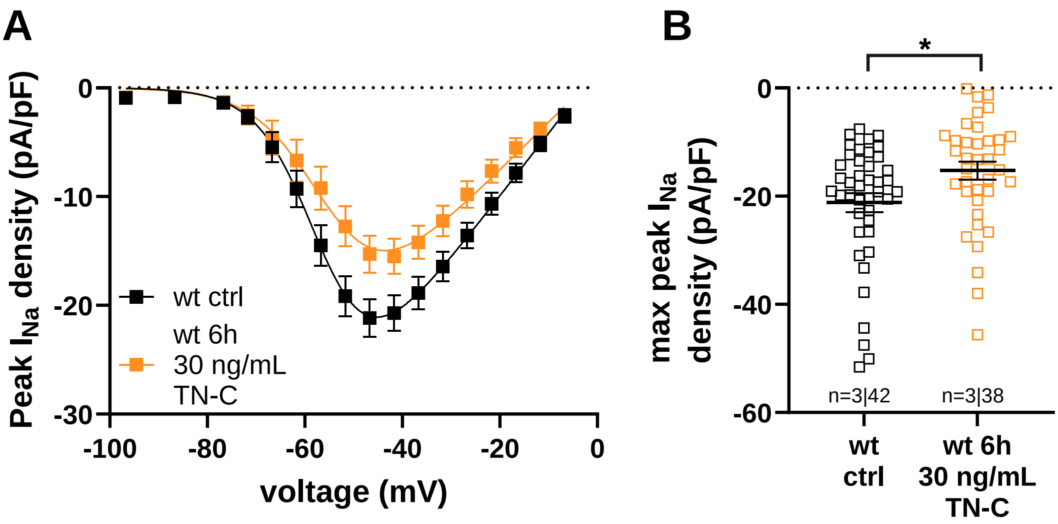

Supplementary Figure 5

Effect of 6 h incubation of wt mouse ventricular cardiomyocytes with human recombinant tenascin-C (hTN-C). (A) Peak I_{Na} density-voltage relationships of untreated and hTN-C (30 ng/mL)-treated wt myocytes. (B) Respective comparison of the peak I_{Na} densities at current maximum (-47 mV); *P<0.05.

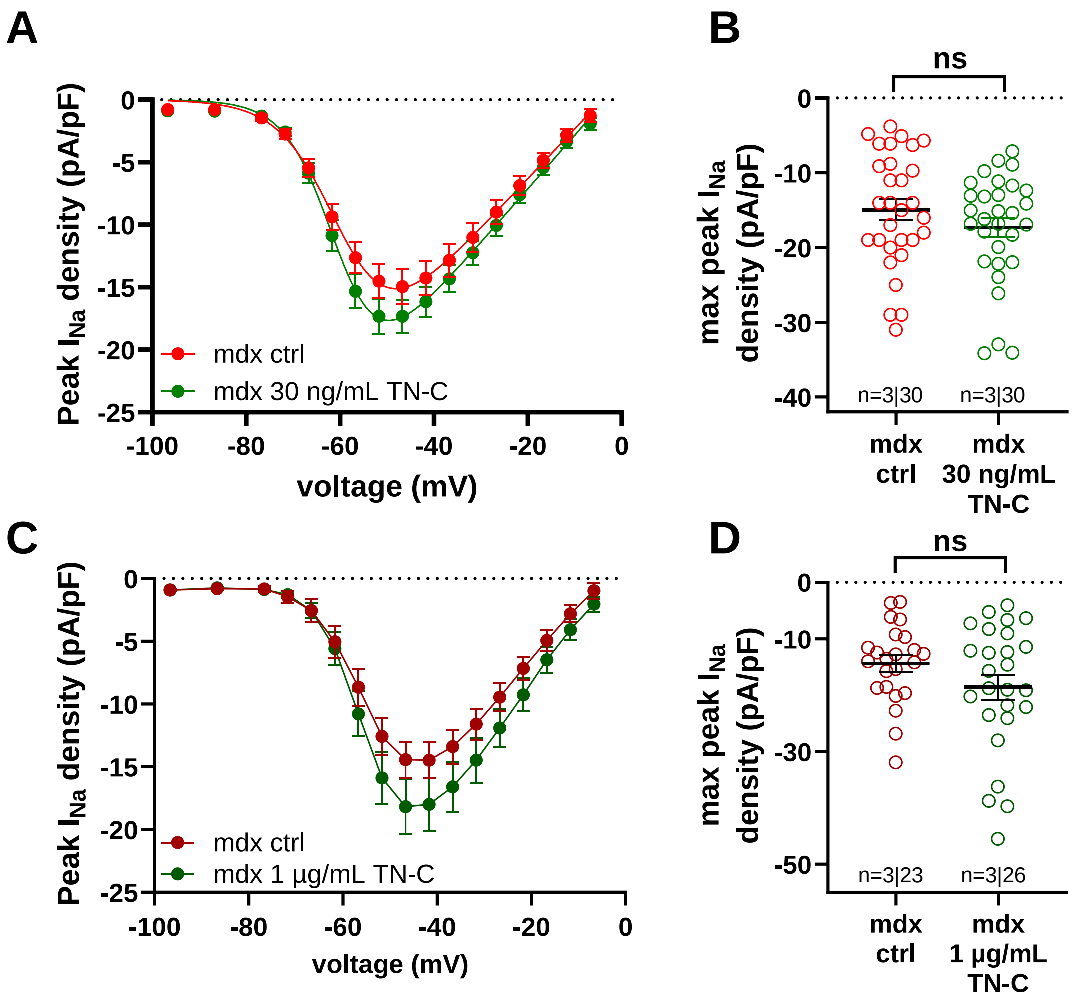

Supplementary Figure 6

Effect of 24 h incubation of dystrophic (*mdx*) mouse ventricular cardiomyocytes with human recombinant tenascin-C (hTN-C). (A) Peak I_{Na} density-voltage relationships of untreated (ctrl) and hTN-C (30 ng/mL)-treated *mdx* myocytes. (B) Respective comparison of the peak I_{Na} densities at current maximum (-47 mV); ns, not significantly different. (C) Peak I_{Na} density-voltage relationships of untreated (ctrl) and hTN-C (1µg/mL)-treated *mdx* myocytes. (D) Respective comparison of the peak I_{Na} densities at current maximum (-47 mV).
